# Supplementary material for: BANKSY unifies cell typing and tissue domain segmentation for scalable spatial omics data analysis
Source: Nat Genet. 2024 Feb 27;56(3):431–41. doi: 10.1038/s41588-024-01664-3 (PMC10937399; doi:10.1038/s41588-024-01664-3)
Supplement: Supplementary file 1 — Supplementary Figs. 1–40, Sections 1–3 and References. [file 41588_2024_1664_MOESM1_ESM.pdf]

nature genetics

Article

https://doi.org/10.1038/s41588-024-01664-3

BANKSY unifies cell typing and tissue domain segmentation for scalable spatial omics data analysis

In the format provided by the authors and unedited

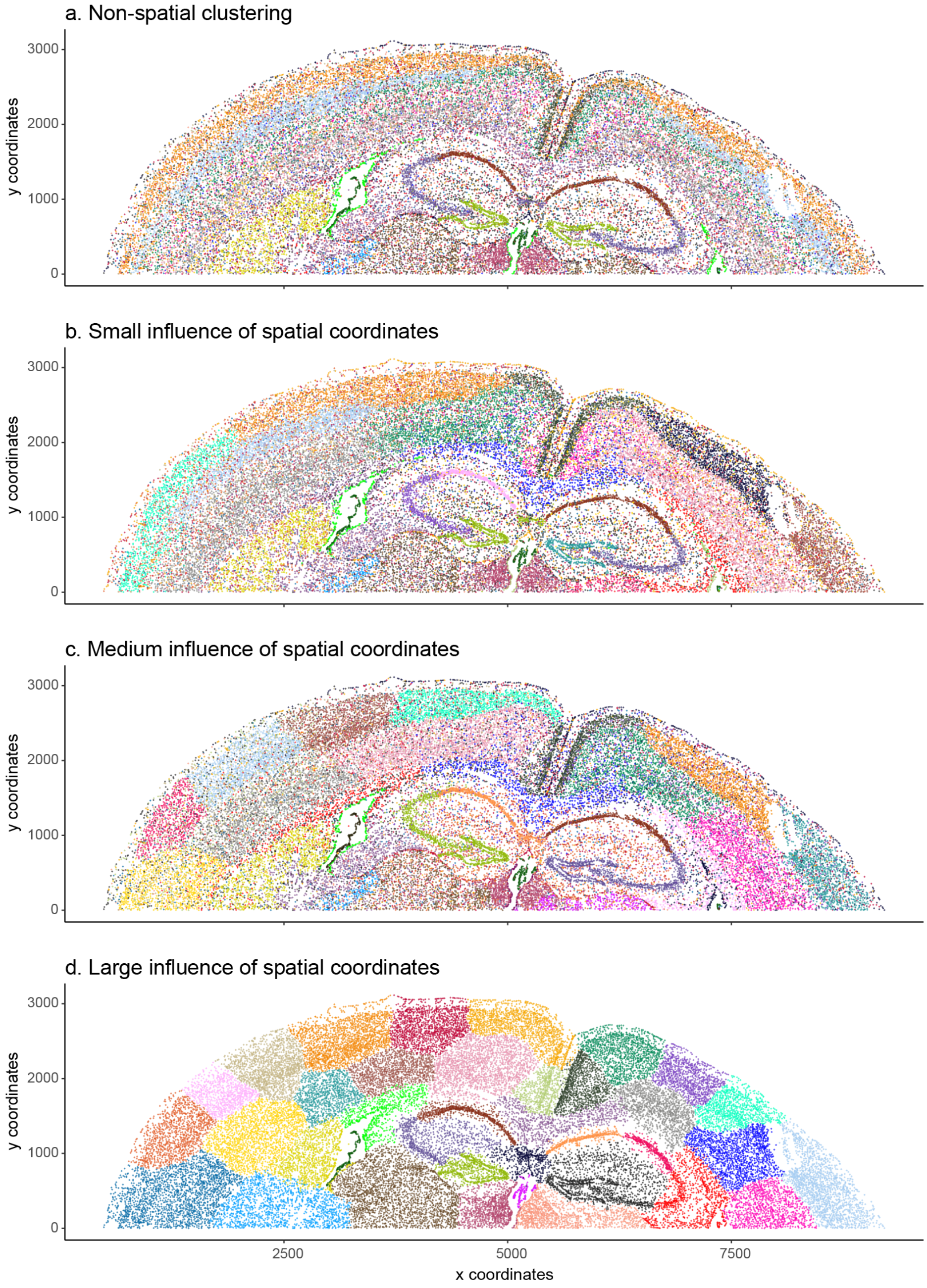

Supp. Fig. 1: Clustering results with the spatial coordinates of cells appended to the gene expression vectors as features (i.e., using the incorrect approach for incorporating spatial information). Coronal section of the mouse brain [1]. (a) Non-spatial clustering. As expected, the left and the right halves of the brain are similar, and are marked by the same clusters. (b) When the spatial locations of cells are appended to the cells' features, following the method described in Fig. 7f of [2], cells that are far apart in physical space are also far apart in feature space, even if they have the same transcriptomic signatures. Thus, the clustering algorithm labels them differently. Spatial coordinates weighting: 1×10^{-6} (small). This effect is stronger when the weighting of the spatial coordinates as features is increased (c: weight = 1×10^{-3} (medium); d: weight = 1×10^{-2} (large)).

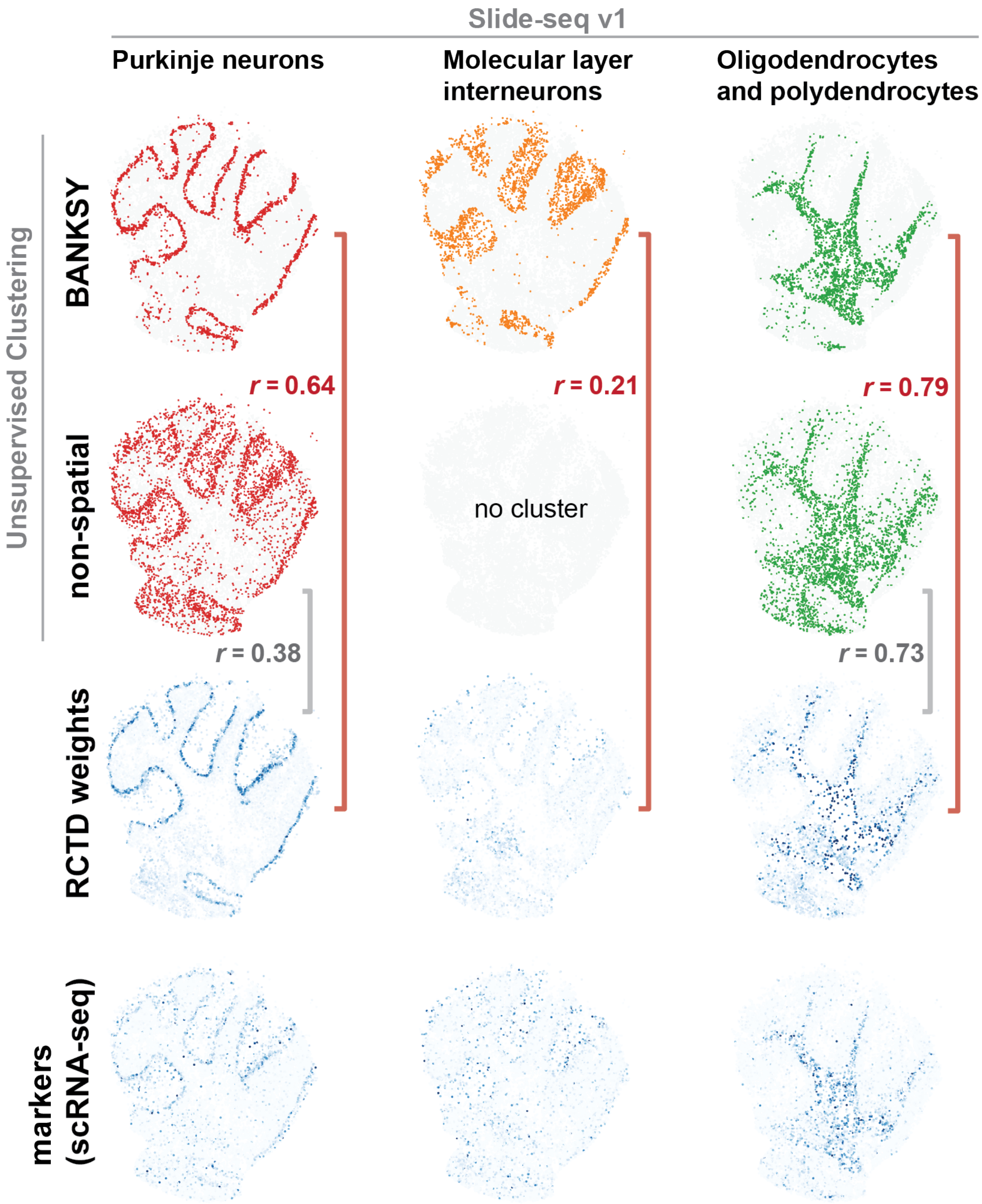

Supp. Fig. 2: Cluster assignments for the individual clusters in the Slide-seq v1 data that were not shown in Fig. 2. As in Fig. 2, bottom two rows show comparison to RCTD weights from corresponding clusters in scRNA-seq reference dataset, and to top DE marker genes from corresponding clusters in the reference dataset (obtained from dropviz.org).

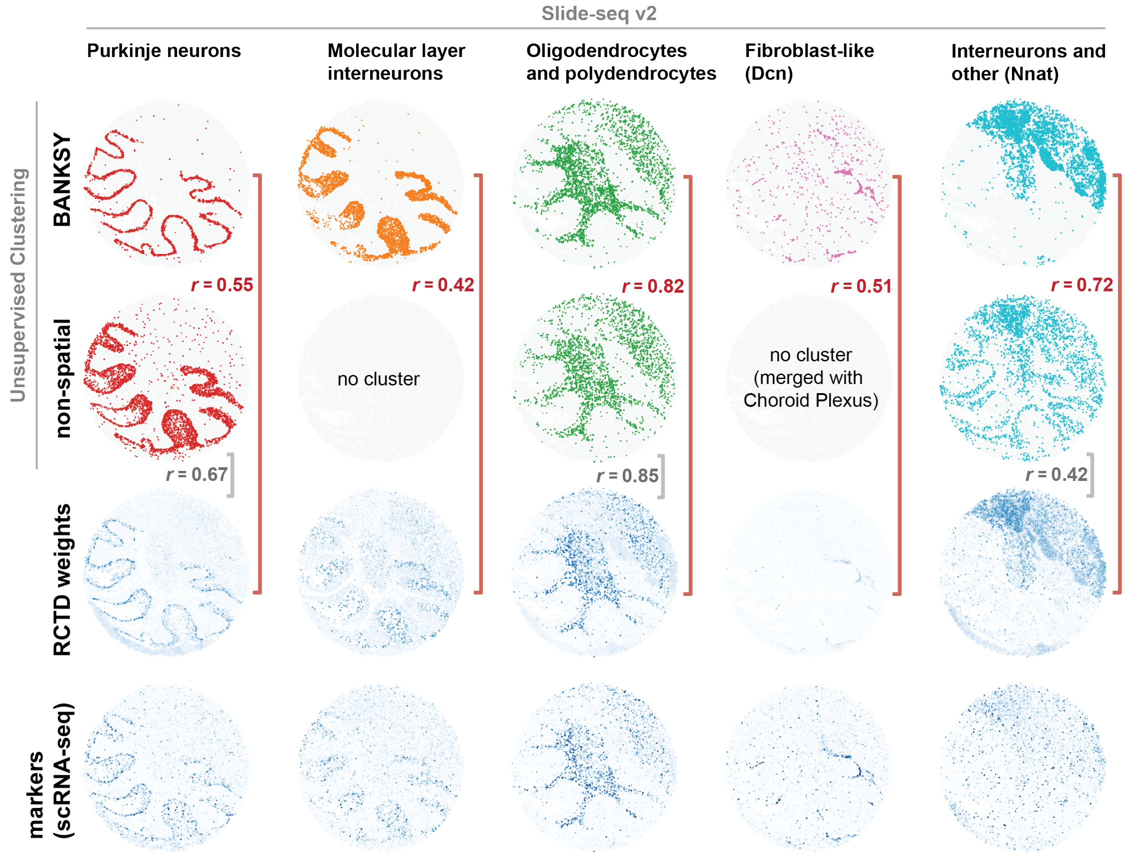

Supp. Fig. 3: Cluster assignments for the individual clusters in the Slide-seq v2 data that were not shown in Fig. 2. As in Fig. 2, lower two rows show comparison to RCTD weights from corresponding clusters in scRNA-seq reference dataset, and to top DE marker genes from corresponding clusters in the reference dataset (obtained from dropviz.org).

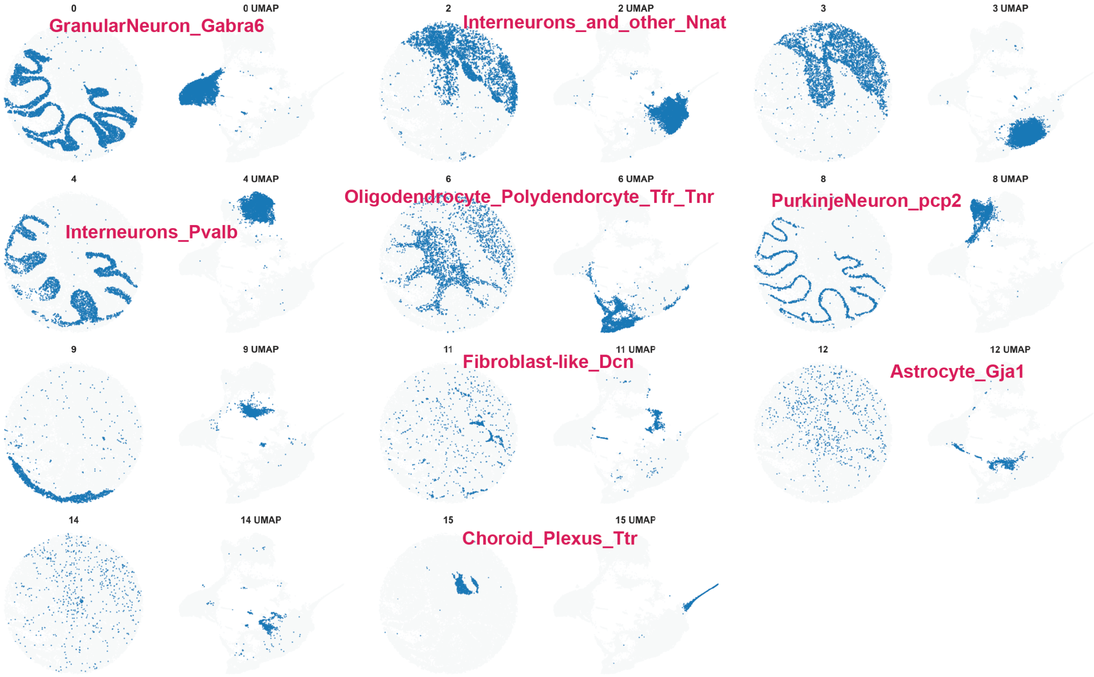

Supp. Fig. 4: All clusters from BANKSY clustering. Clusters identifed as matching cell-type signatures in reference scRNA-seq dataset (from dropviz.org) are labeled in red.

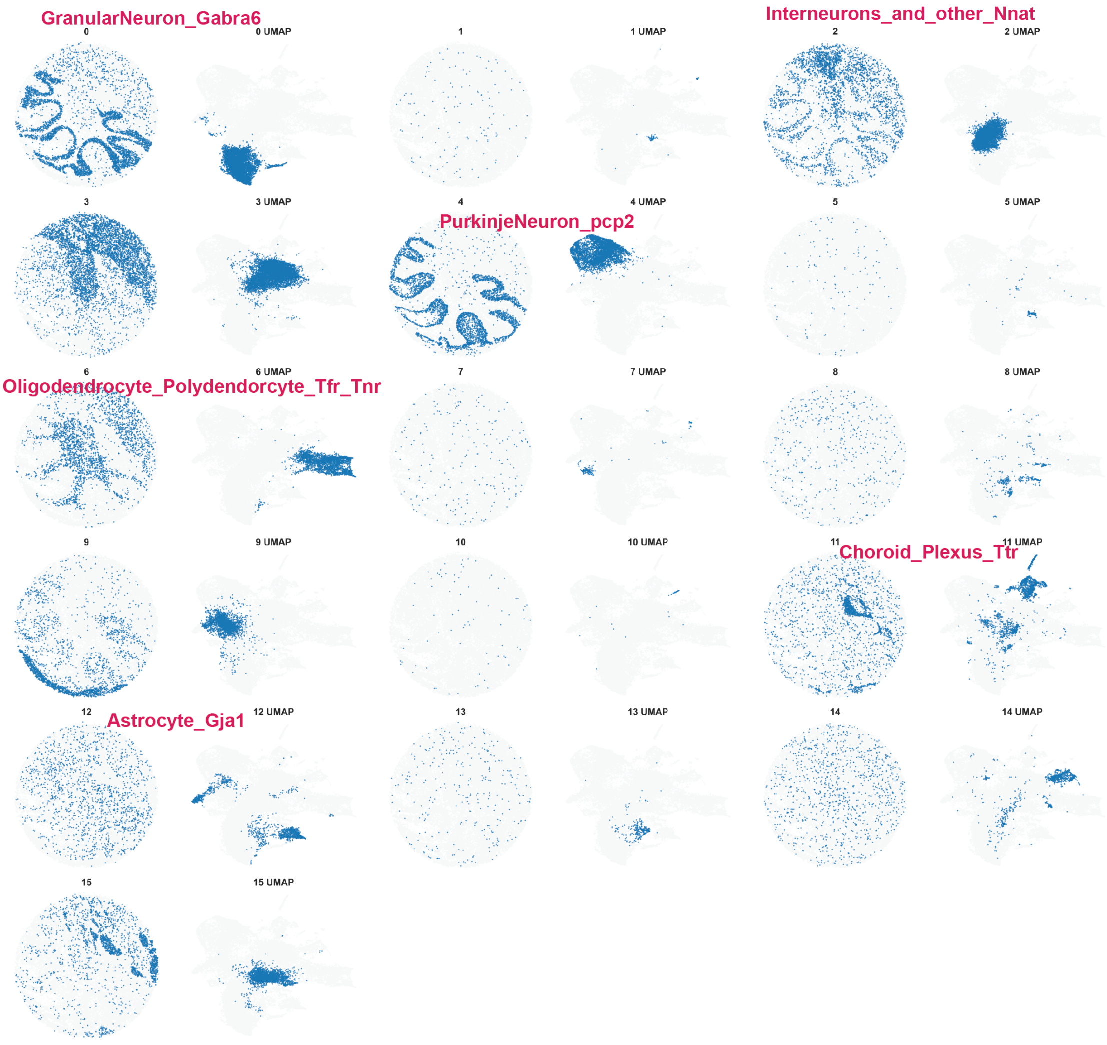

Supp. Fig. 5: All clusters from non-spatial clustering. Clusters identifed as matching cell-type signatures in reference scRNA-seq dataset (from dropviz.org) are labeled in red.

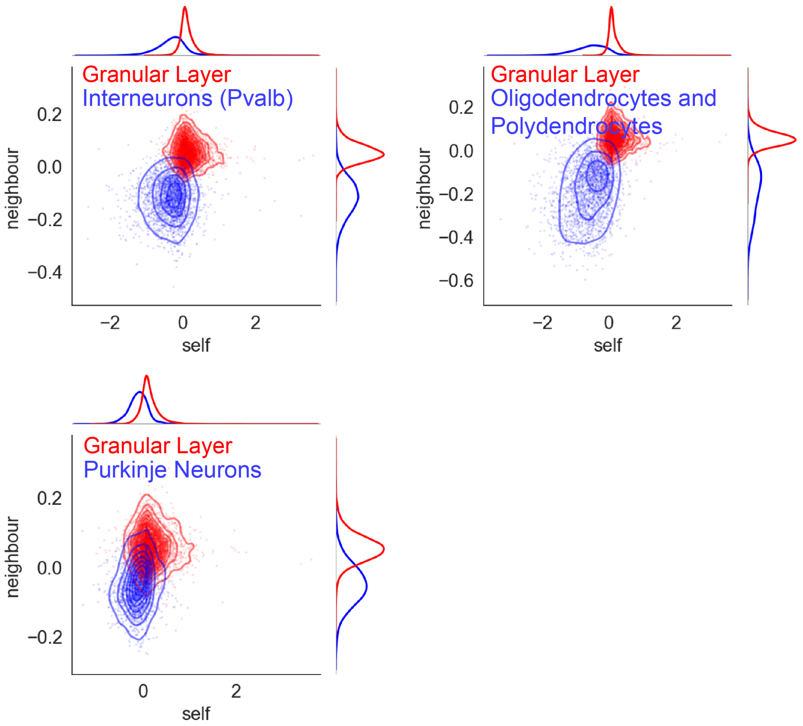

Supp. Fig. 6: Plot of the difference in metagene expression (mean of top 20 DE genes, see Methods Section 4.3) in the granular layer cluster vs other clusters in Slide-seq v1, comparing each other cluster (blue) to the granular layer (red). X-axis shows cells' own expression. Y-axis shows average neighbor expression.

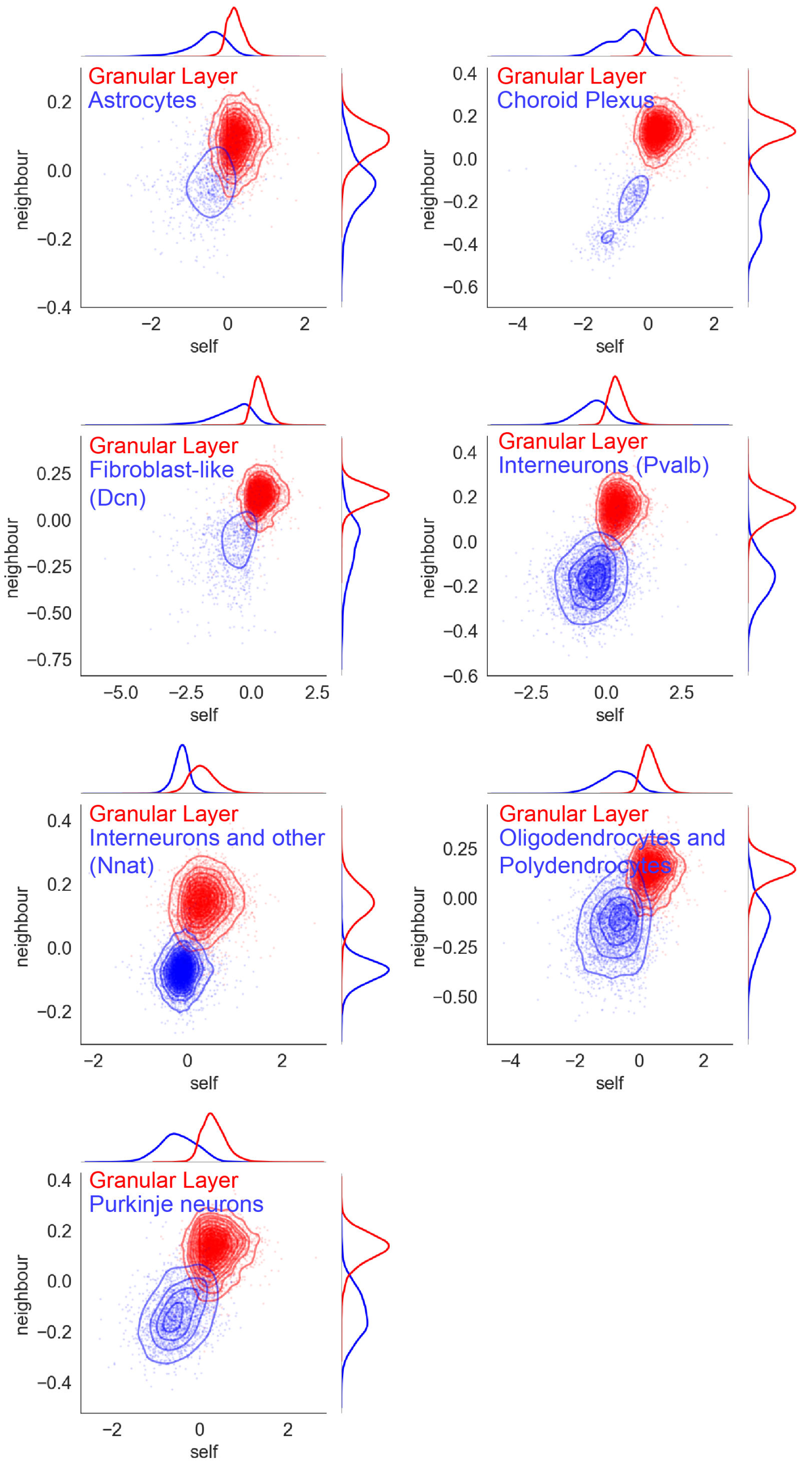

Supp. Fig. 7: Plot of the difference in metagene expression (mean of top 20 DE genes,see Methods Section 4.3) in the granular layer cluster vs other clusters in Slide-seq v2, comparing each other cluster (blue) to the granular layer (red). X-axis shows cells' own expression. Y-axis shows the average neighbor expression.

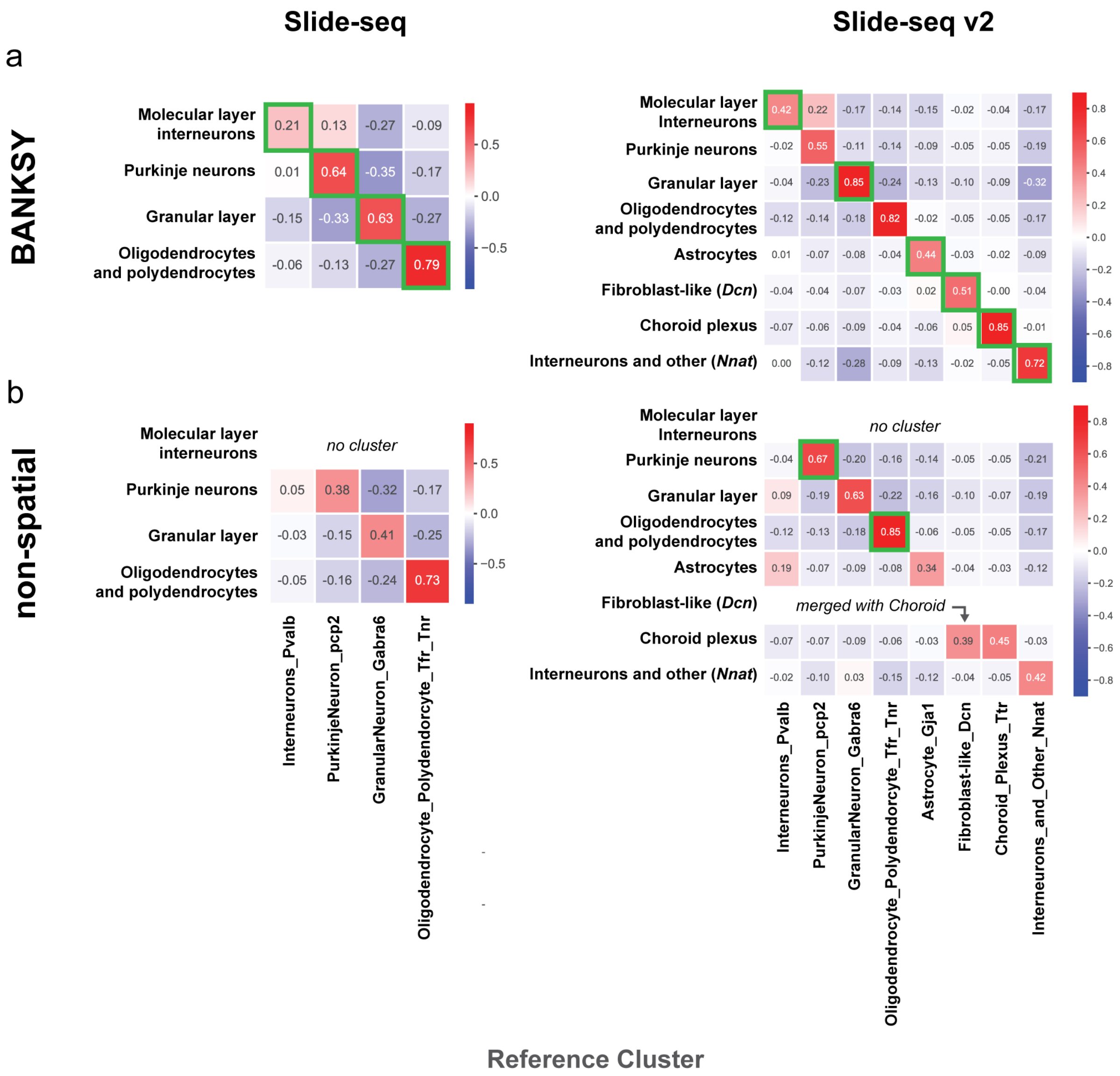

Supp. Fig. 8: (a) Point-biserial correlation (r) between each BANKSY cluster and RCTD weights from the corresponding reference cluster, for both Slide-seq and Slide-seq v2. (b) Same as (a), for non-spatial clusters. Green boxes indicate higher r values. All correlations along the diagonals are significant (associated p-values, two-sided, uncorrected ≤ 0.05).

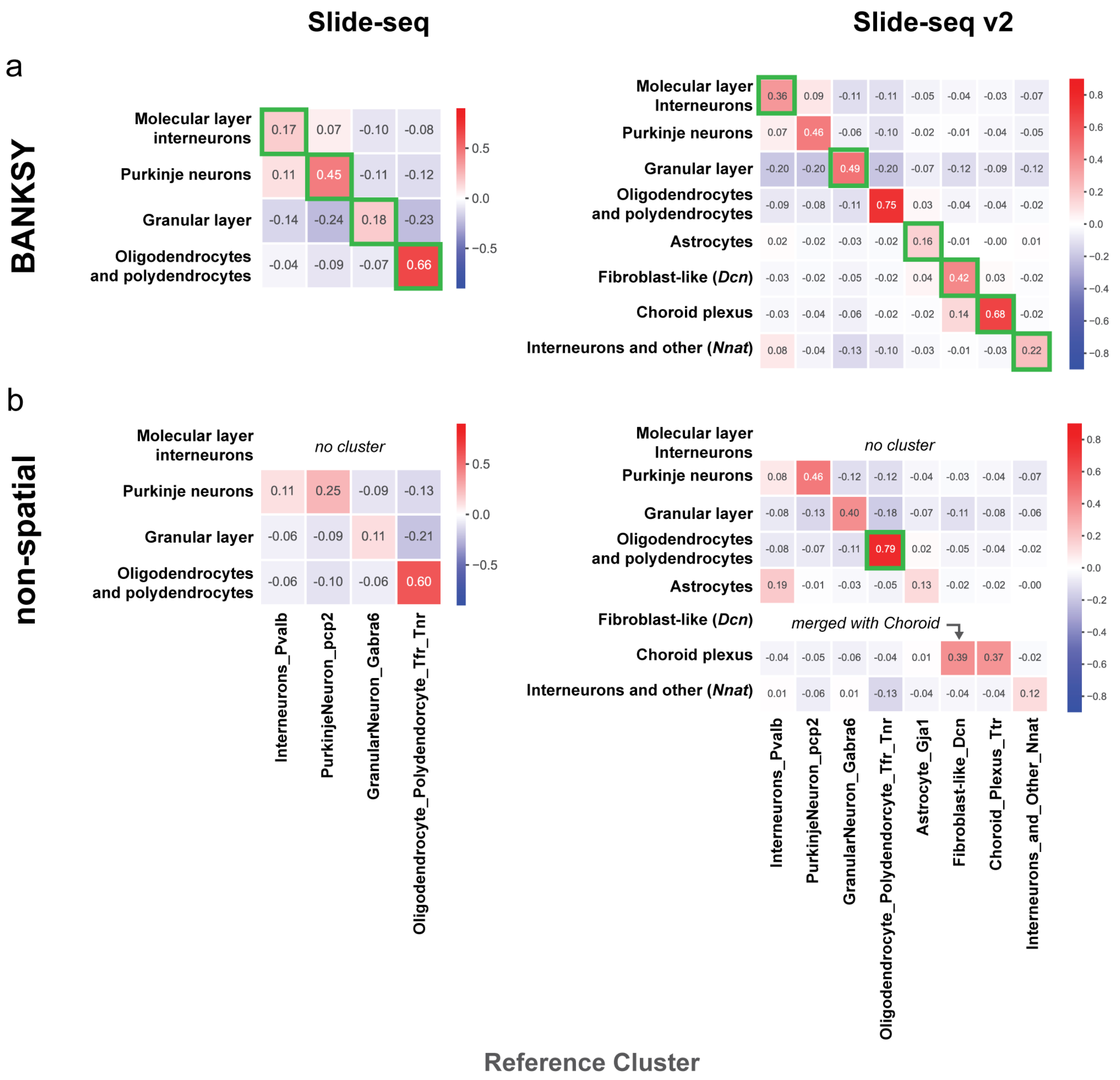

Supp. Fig. 9: (a) Point-biserial correlation (r) between each BANKSY cluster and metagene (mean expression of top DE genes in reference cell type) from the corresponding reference cluster, for both Slide-seq and Slide-seq v2. (b) Same as (a), for non-spatial clusters. Green boxes indicate higher r values. All correlations along the diagonals are significant (associated p-values, two-sided, uncorrected ≤ 0.05).

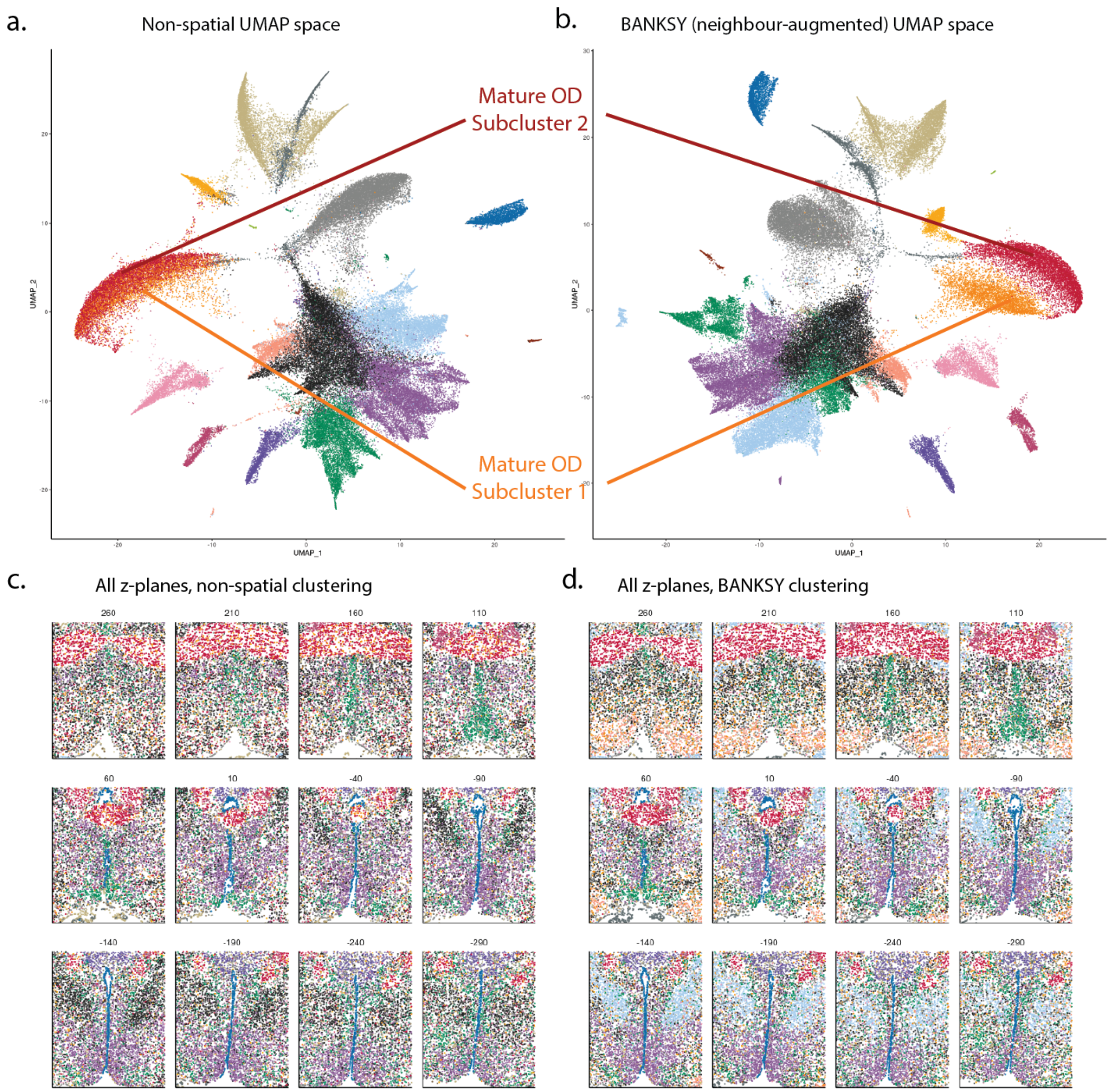

Supp. Fig. 10: (a) UMAP representation of the cells in the own expression feature space versus those in the BANKSY embedding space, colored by BANKSY cluster labels. This shows that the mature oligodendrocyte subclusters were mixed in the own-expression (conventional) feature space, but separated out in the BANKSY embedding space (orange and red clusters). (c, d) The tissue maps show all twelve z-planes with cells colored by both non-spatial clustering (c) and BANKSY clustering (d).

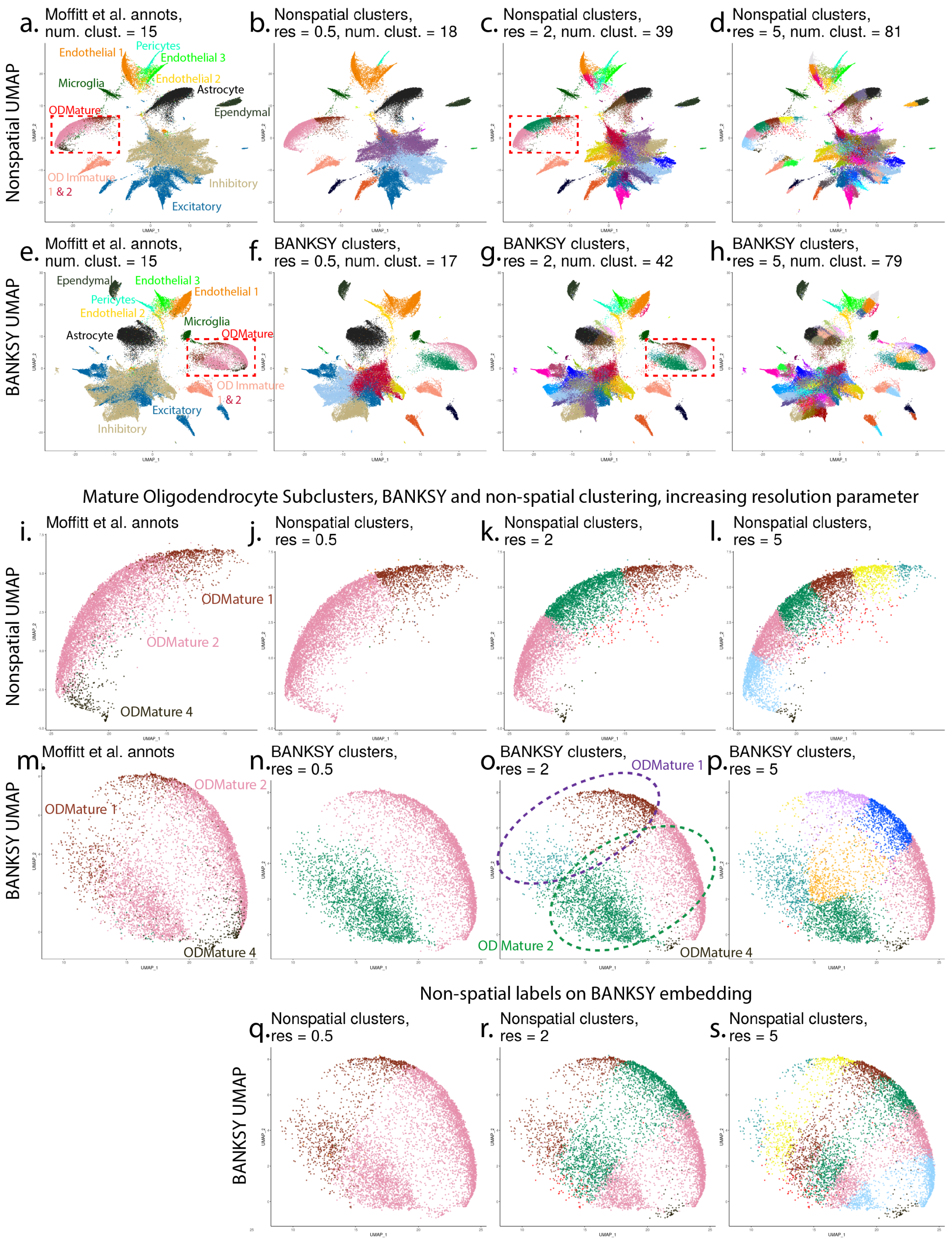

Supp. Fig. 11: Mature oligodendrocytes shown on both the non-spatial and BANKSY UMAP coordinates. (a-d) Non-spatial UMAP coordinates, all cells. (a) Moffitt et al.'s labels. (b-d) Our non-spatial clustering ($\lambda = 0$) labels at increasing clustering resolutions. (e-h) BANKSY embedding UMAP coordinates. (e) Moffitt et al.'s labels. (f-h) BANKSY ($\lambda = 0.2$) labels at increasing clustering resolutions. (i-l): Boxed region in panel a, with Moffitt et al.'s labels (panel i), and our non-spatial labels at increasing clustering resolutions. (m-p) Boxed region in panel e, with Moffitt et al.'s labels (panel m), and BANKSY's labels at increasing clustering resolutions. In particular, panels m and n show that the Moffitt et al. non-spatial labeling is along a complementary axes relative to the BANKSY split, and panel o shows that at higher resolutions, BANKSY is able to split the cells along *both* axes. (q-s) Non-spatial labels on the BANKSY embedding UMAPs showing that non-spatial clustering at higher resolutions cannot capture the BANKSY split.

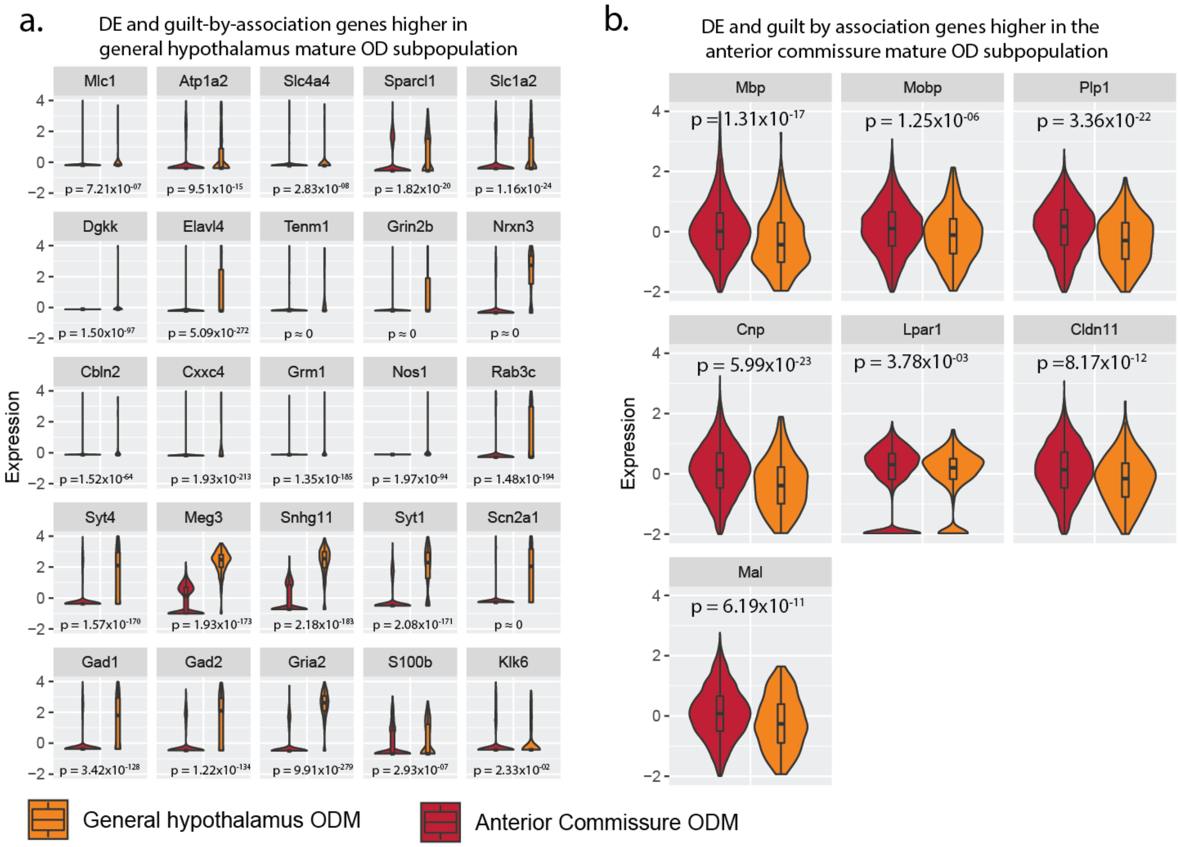

Supp. Fig. 12: scRNA-seq corroboration of the mature oligodendrocyte (ODM) subclusters identified by BANKSY in the mouse hypothalamus MERFISH dataset (Fig. 3). Distribution of scRNA-seq expression levels of MERFISH DE genes and their top scRNA-seq guilt-by-association neighbors in mature oligodendrocytes of the anterior commissure (red, MOD-wm, n = 6333 cells) and general hypothalamus (orange, MOD-gm, n = 278 cells). (a) Genes with lower expression in anterior commissure. Center line: median, height of box: interquartile range (IQR), whiskers:1.5×IQR. (b) Genes with higher expression in the anterior commissure. Center line: median, height of box: interquartile range (IQR), whiskers:1.5×IQR. P-values: one-sided unpaired Wilcoxon rank-sum test.

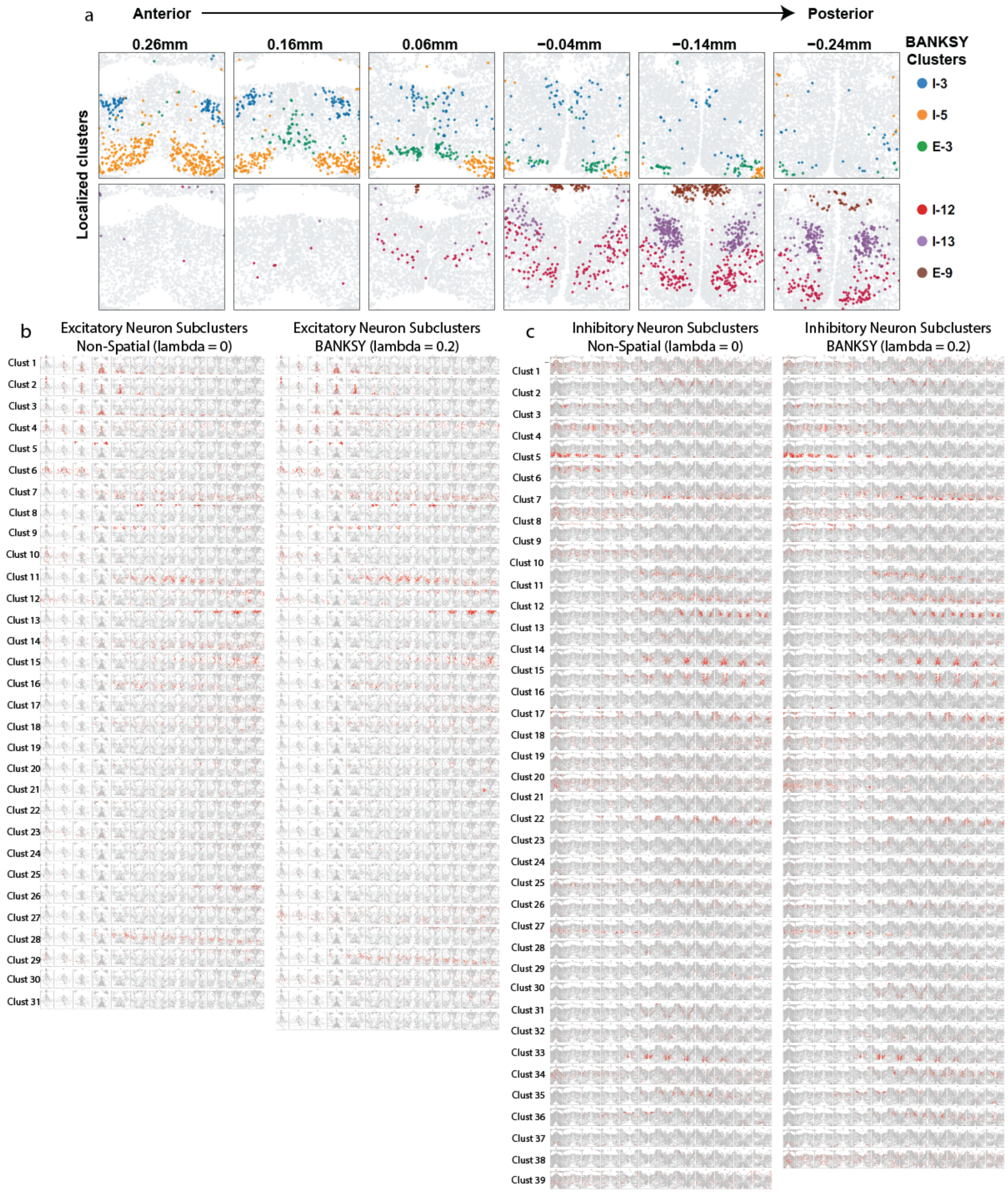

Supp. Fig. 13: Excitatory and inhibitory neuronal subtypes in the MERFISH mouse hypothalamus dataset. (a) BANKSY found the 6 spatially localized neuronal subtypes that were also highlighted by the authors of the original study [3] in their main Fig. 5a. (b, c) 70 neuronal subtypes identified by non-spatial and BANKSY iterative clustering, similar to Supp. Fig. S17 in the original study.

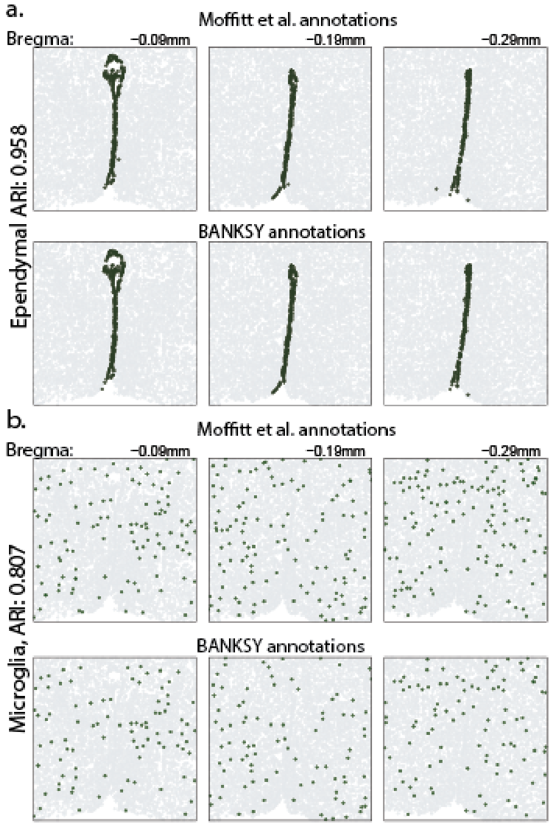

Supp. Fig. 14: BANKSY's spatial clustering continues to identify rare cell types identified by the original study [3]. (a) Single cell thick layer of ependymal cells in the third ventricle (spatially localized population, comprising 2.41% of cells (ARI between the Moffitt et al. annotations and BANKSY: 0.958). (b) The spatially dispersed microglia population, comprising 2.12% of cells (ARI: 0.807). See Supp. Section 3.

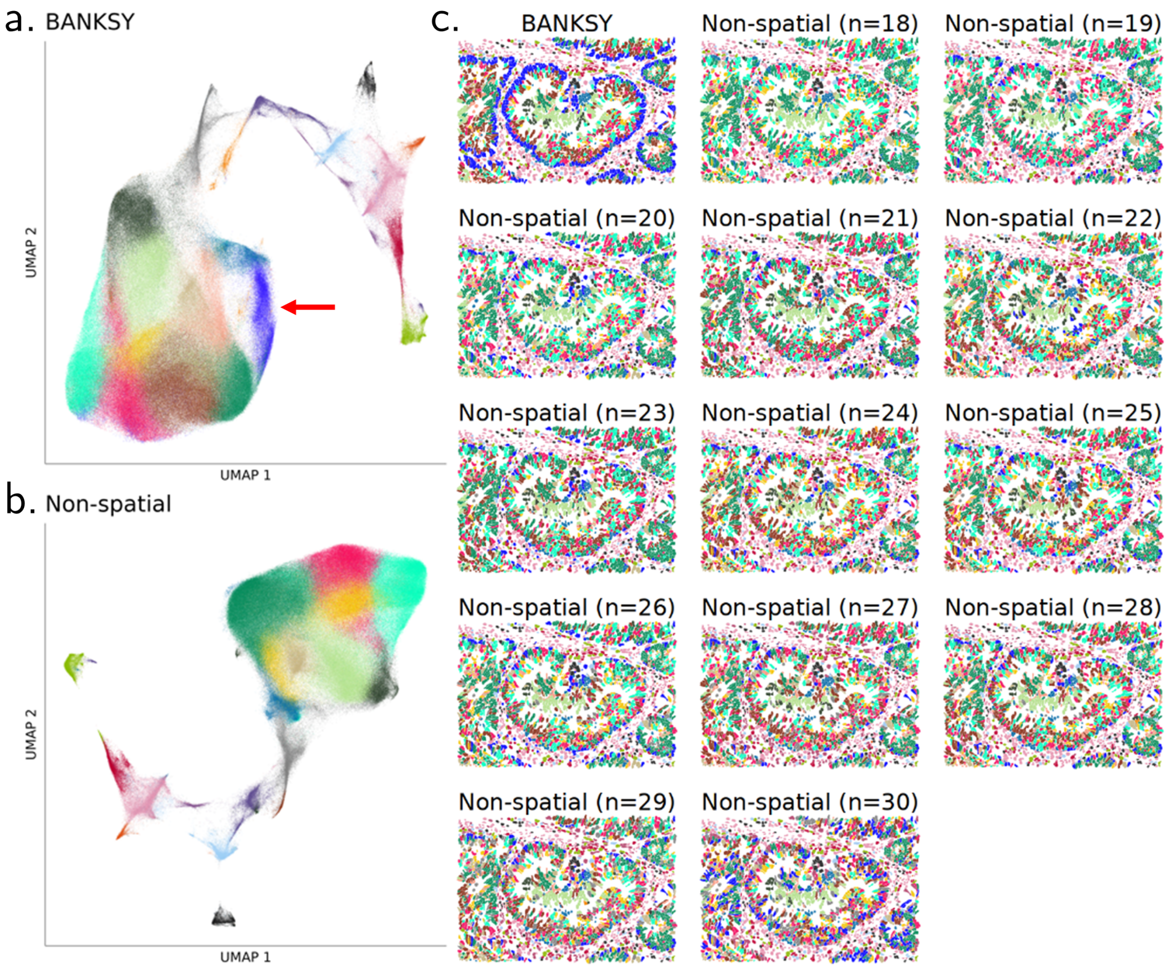

Supp. Fig. 15: Comparison of BANKSY and non-spatial clustering on MERSCOPE colorectal tumor data. a) BANKSY UMAP, with a unique cycling cell population indicated by a red arrow. b) Non-spatial UMAP. c) Spatial plots illustrating non-spatial clusters at increasing resolutions, zoomed in to a single gland to visualize individual cells.

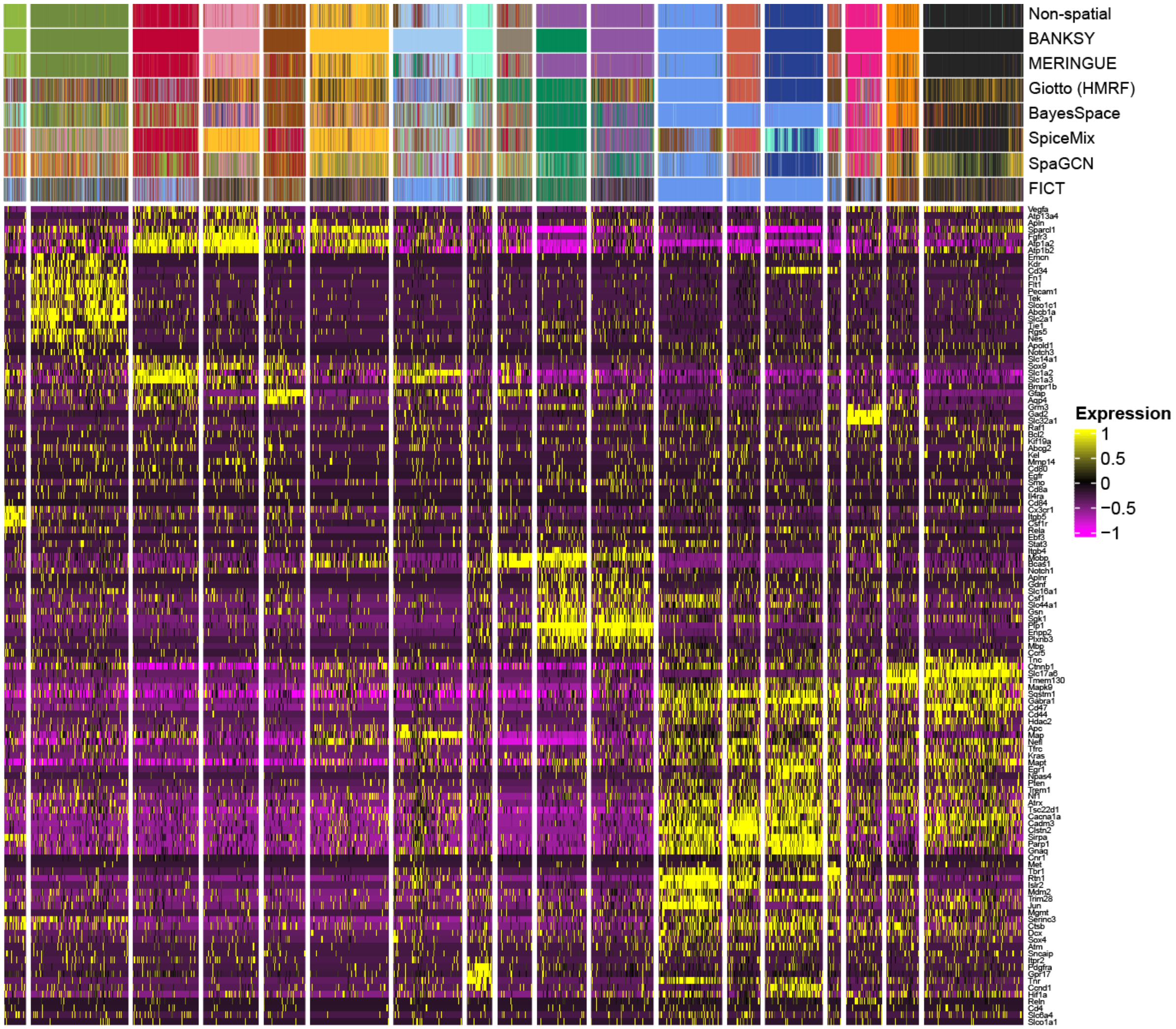

Supp. Fig. 16: Heatmap showing the gene expression for the VeraFISH mouse hippocampus data in Fig. 4, with annotation bars for all eight methods tested.

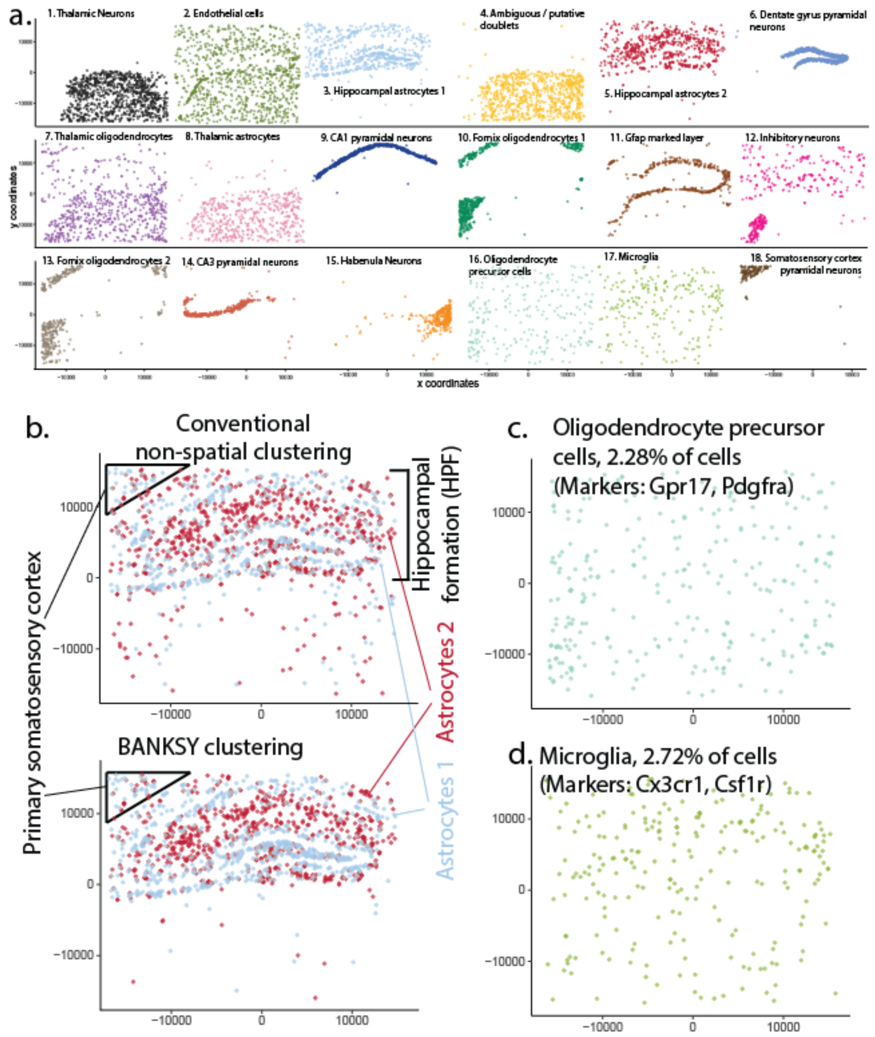

Supp. Fig. 17: Spatial distributions of VeraFISH mouse brain data clusters (Fig. 4), with annotations based on key markers: 1–thalamic neurons (Slc17a6); 2–endothelial cells (Pecam1, Slc2a1); 3–hippocampal astrocytes 1 (dentate gyrus enriched, Slc1a2); 4–Ambiguous/putative doublets (scattered expression of Mobp/Bcas1 (oligodendrocytes), Nefl (neurons), and Atp1a2 (astrocytes)); 5–hippocampal astrocytes 2 (CA3 enriched, Sox8); 6–dentate gyrus neurons; 7–thalamic oligodendrocytes (Plp1, Mobp, Mbp); 8–thalamic astrocytes (Fgfr3); 9–CA1 neurons (Egr1, [4]); 10–fornix oligodendrocyts 1 (Plp1, Mobp, Mbp); 11–Gfap marked layer (possible astrocytes [5, 6]); 12–Inhibitory neurons (Gad2, Slc32a1); 13–fornix oligodendrocytes 2 (Plp1, Mobp); 14–CA3 neurons (Cadm3 [7]); 15–habenula neurons (Slc17a6, Tmem130); 16–oligodendrocyte precursor cells (OPCs: Gpr17, Pdgfra); 17–microglia (Cx3cr1, Csf1r); 18–somatosensory cortex neurons (Tbr1, [8]). (b) two closely related astrocyte subtypes, intermixed within the hippocampal formation and primary somatosensory cortex of mouse brain. Upper panel: non-spatial cluster labels. Lower panel: BANKSY cluster labels. Further examples of distinct cell types that are spatially intermingled, and remain separable by BANKSY can be found in panel a: thalamic neurons, endothelial cells, thalamic oligodendrocytes, thalamic astrocytes, OPCs, and microglia. (c, d) Examples of spatially dispersed cell-types with distinctive marker gene expression identified by BANKSY in the VeraFISH data: OPCs making up 2.28% of cells and microglia making up 2.72% of cells. See Supp. Section 3.

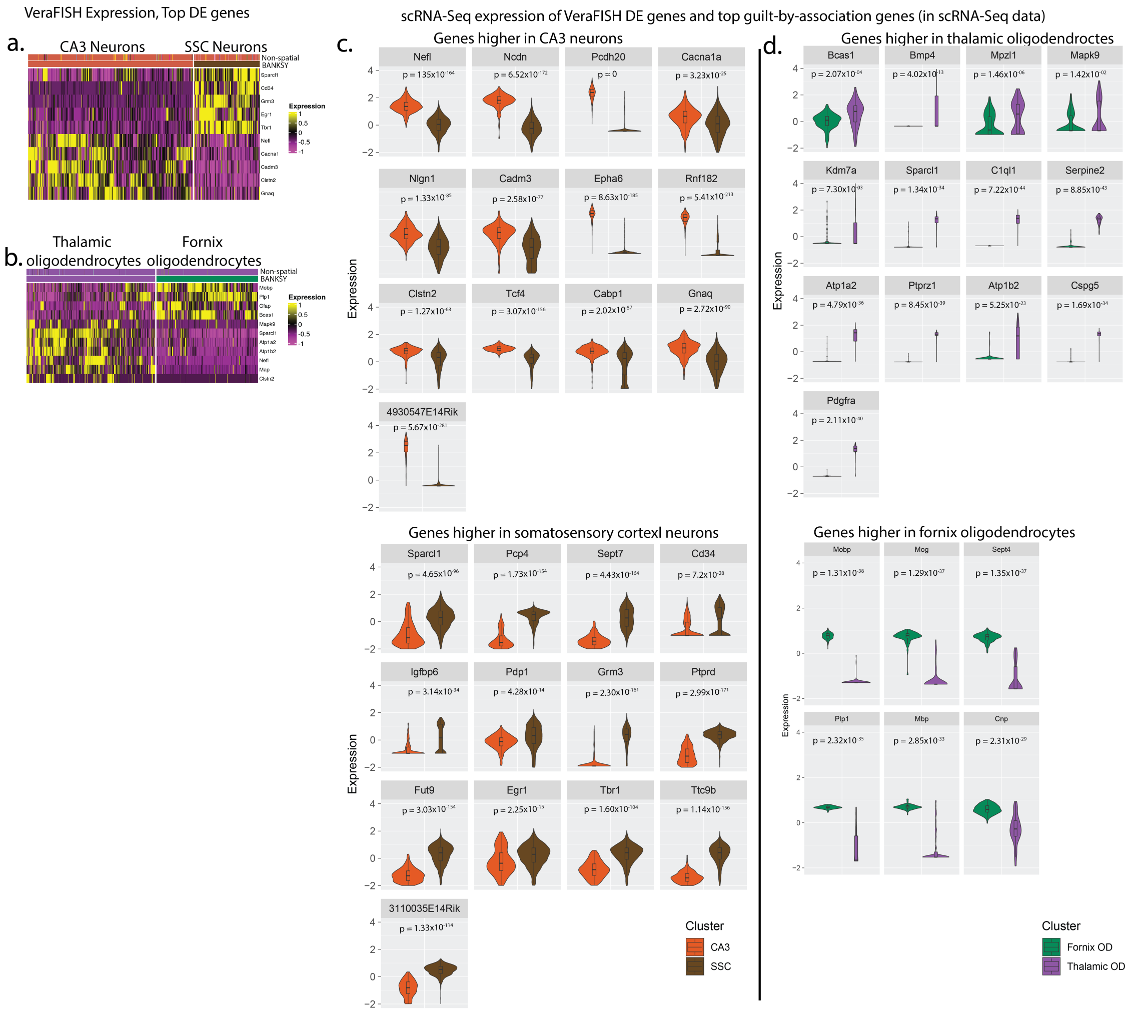

Supp. Fig. 18: scRNA-seq corroboration of the neuronal and oligodendrocyte subpopulations identified by BANKSY in mouse hippocampus VeraFISH data. (a) Heatmap showing the top DE genes between CA3 neurons and somatosensory cortex neurons. (b) Similar heatmap showing thalamic and fornix oligodendrocyte DE genes. (c) Violin plots showing the expression of top marker and guilt-by-association genes in the two neuronal (CA3 neurons, n = 322 cells; SSC neurons, n = 2064 cells) and (d) oligodendrocyte (thalamic oligos, n = 87 cells; fornix oligos, n = 144 cells) subpopulations. Top: genes higher in CA3 neurons and thalamic oligodendrocytes. Bottom: genes higher in somatosensory cortex neurons and fornix oligodendrocytes. Center line: median, height of box: interquartile range (IQR), whiskers:1.5×IQR. P-values: one-sided unpaired Wilcoxon rank-sum test.

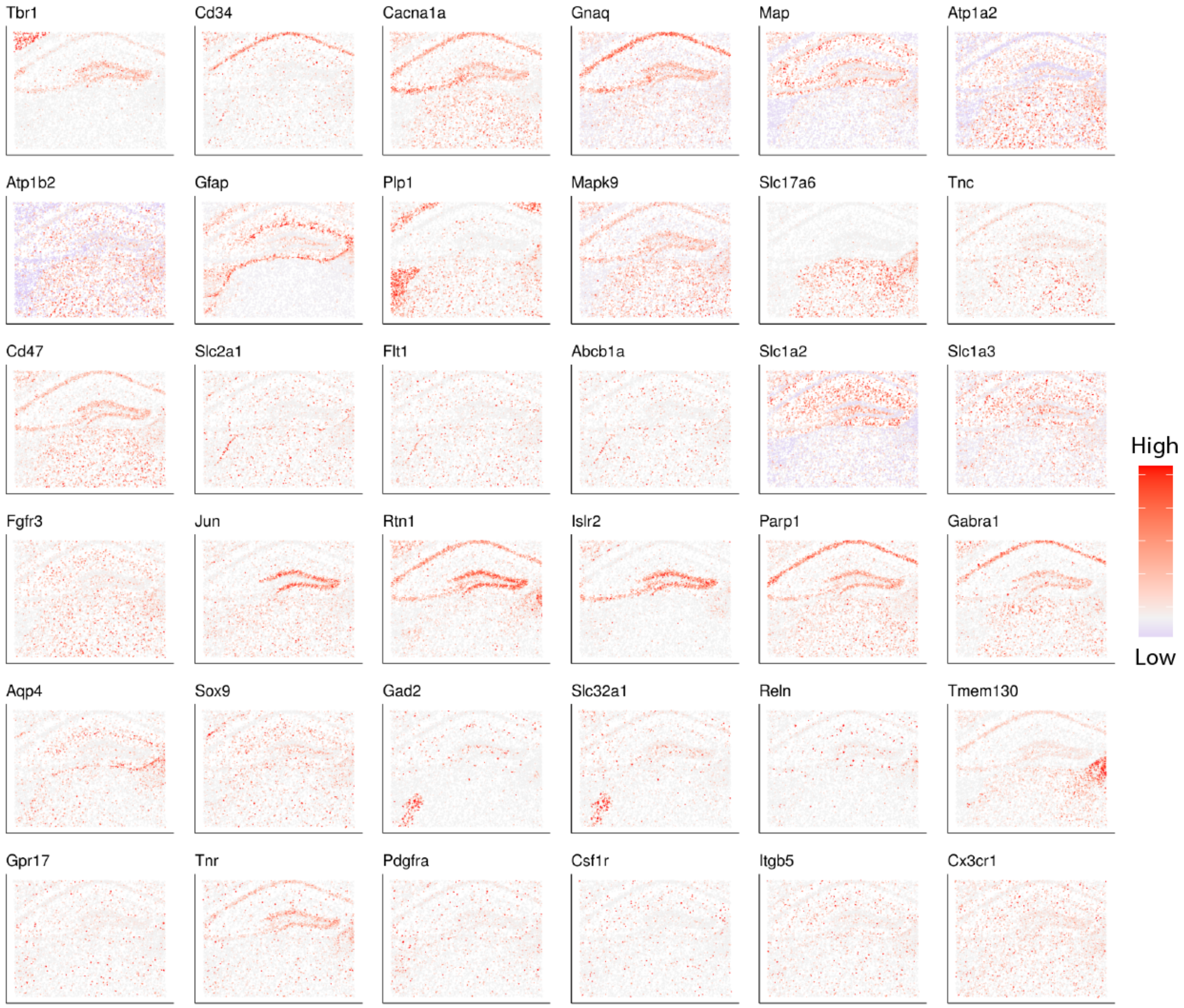

Supp. Fig. 19: Spatial distributions of additional DE genes that distinguish the clusters in Fig. 4. Red: high expression, White/purple: low expression.

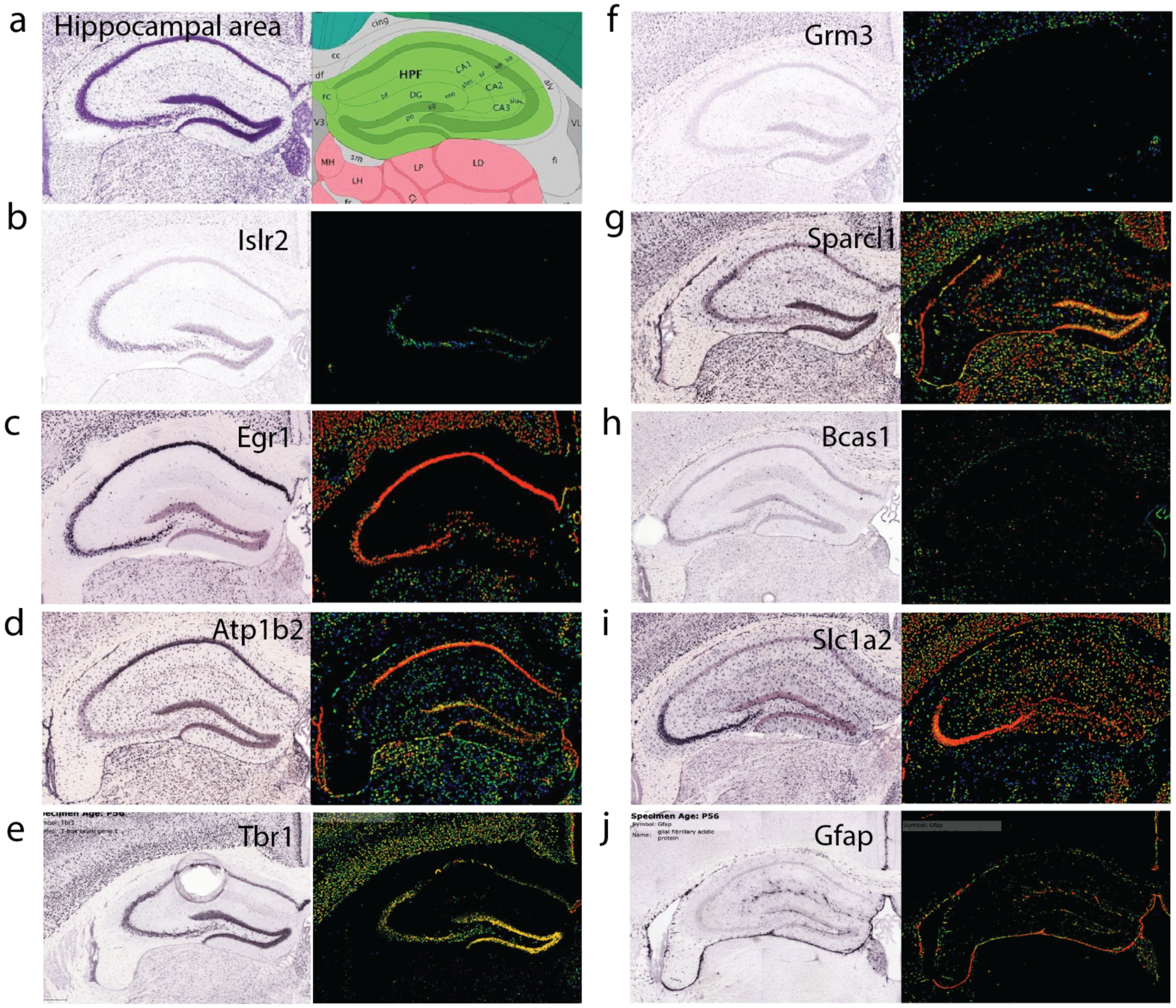

Supp. Fig. 20: Allen Mouse Brain Atlas images [9, 10] showing ISH and expression levels for some of the DE genes in Fig. 4. (a) Schematic labelling the main regions in the hippocampus. (b-j) ISH images and expression levels for genes in this region.

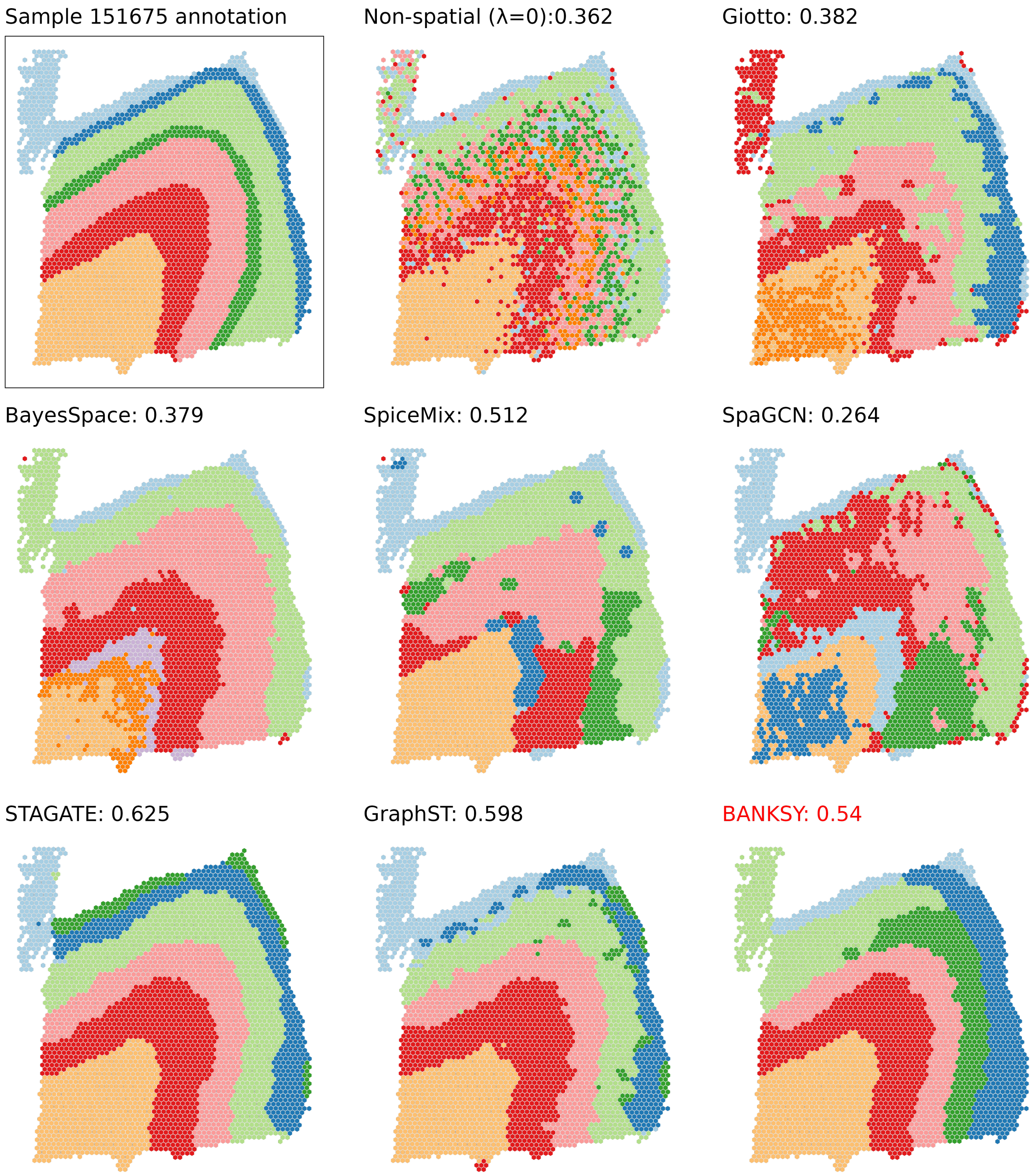

Supp. Fig. 21: Qualitative cluster comparison for sample 151675, which is different from the sample shown in the main text (sample 151673).

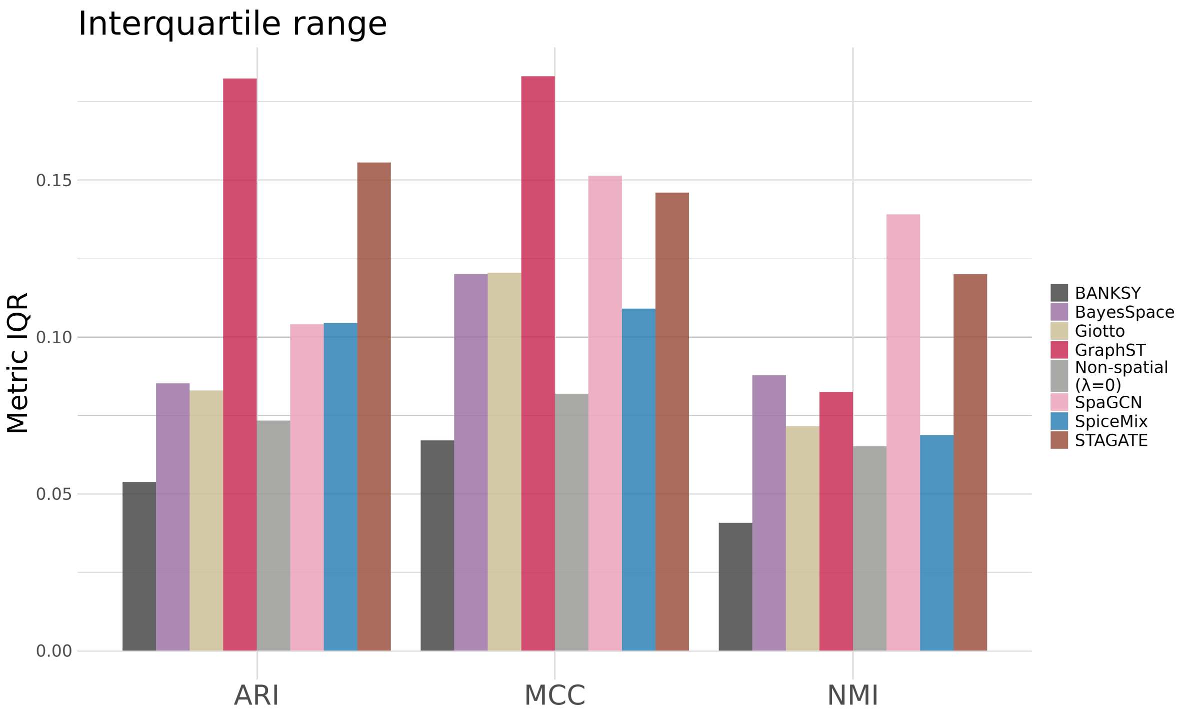

Supp. Fig. 22: Inter-quartile range of accuracy scores (three metrics) across 12 DLPFC samples for each method benchmarked. We used three metrics to quantify performance relative to the expert-annotated ground truth: adjusted Rand index (ARI), normalized mutual information (NMI) and Matthews correlation coefficient (MCC).

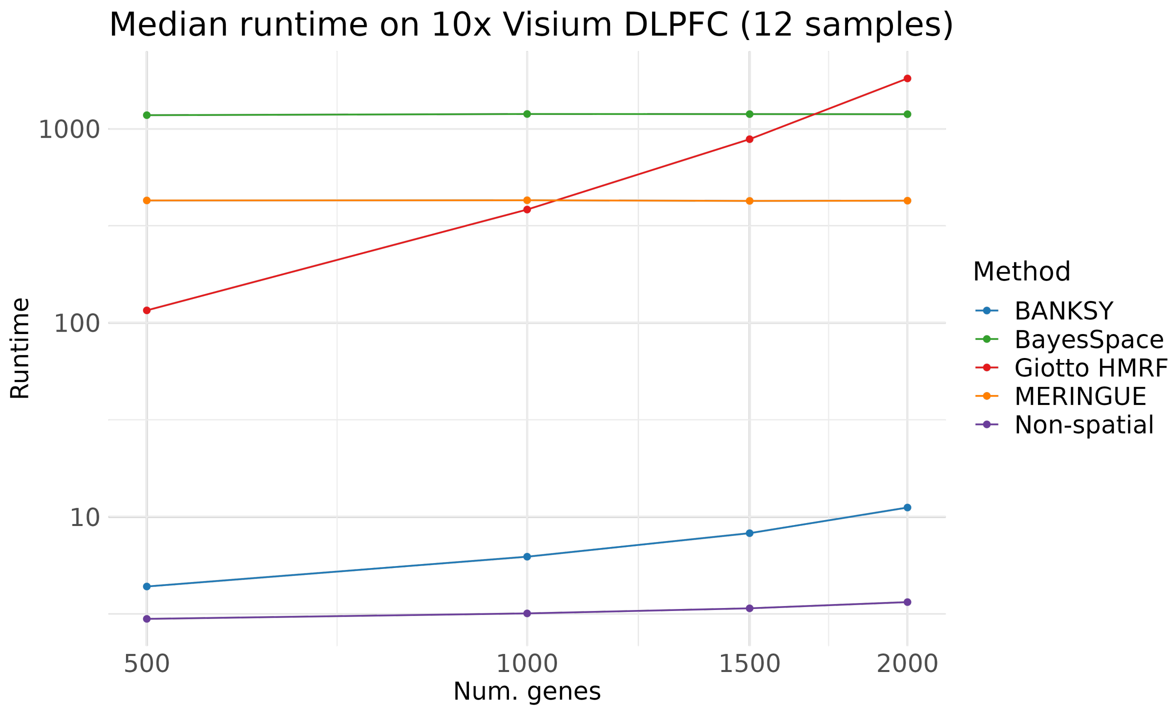

Supp. Fig. 23: Runtime for the DLPFC dataset as a function of the number of genes used for clustering.

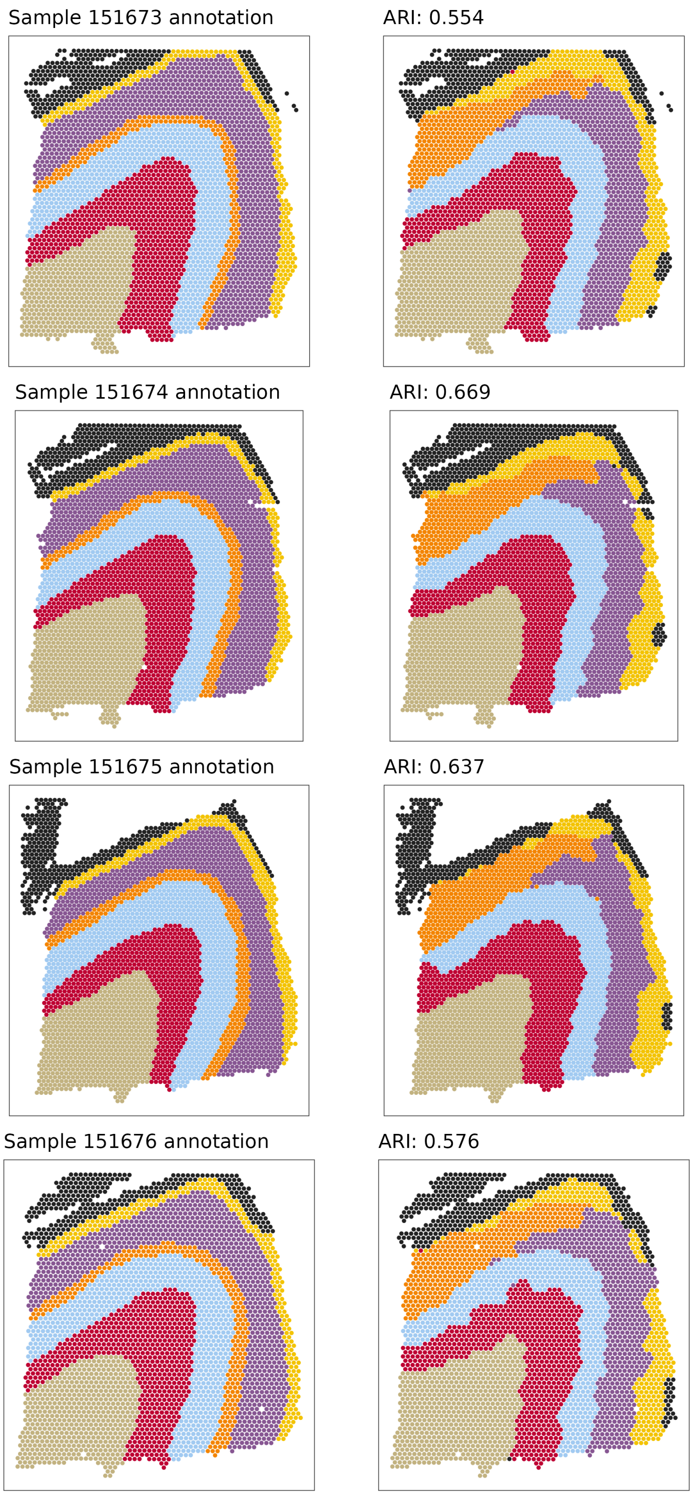

Supp. Fig. 24: Multi-sample analysis on 4 DLPFC Visium datasets from the same subject. Samples are z-transformed separately and jointly clustered. Left: sample annotations. Right: BANKSY clusters.

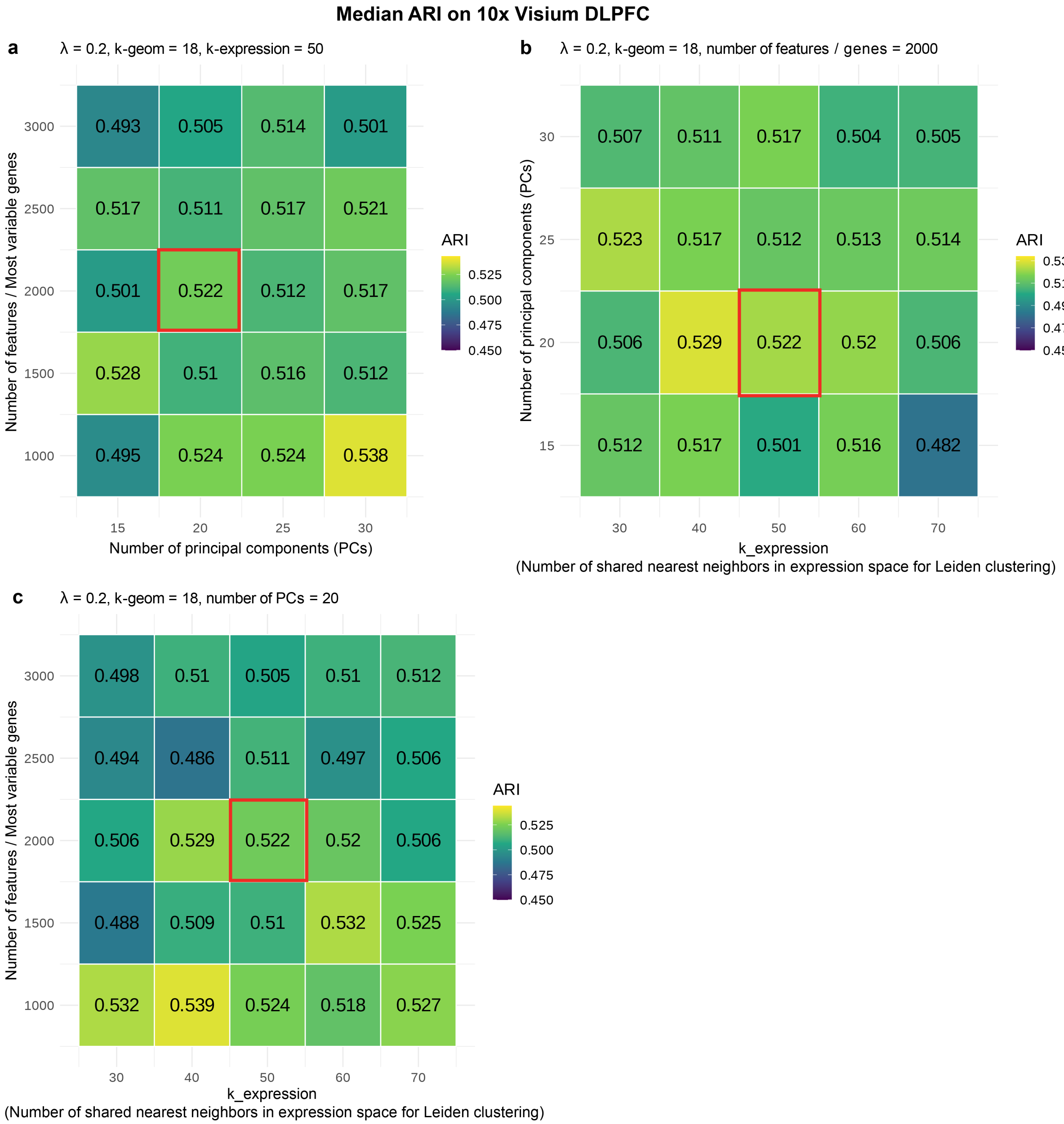

Supp. Fig. 25: Parameter sweep on the 10X Visium DLPFC dataset, varying the number of features used (highly variable genes), number of principal components (PCs) and k_{expr}. (a) k_{expr} held constant. (b) Number of highly variable genes held constant. (c) Number of principal components (PCs) held constant. Red boxes show default values.

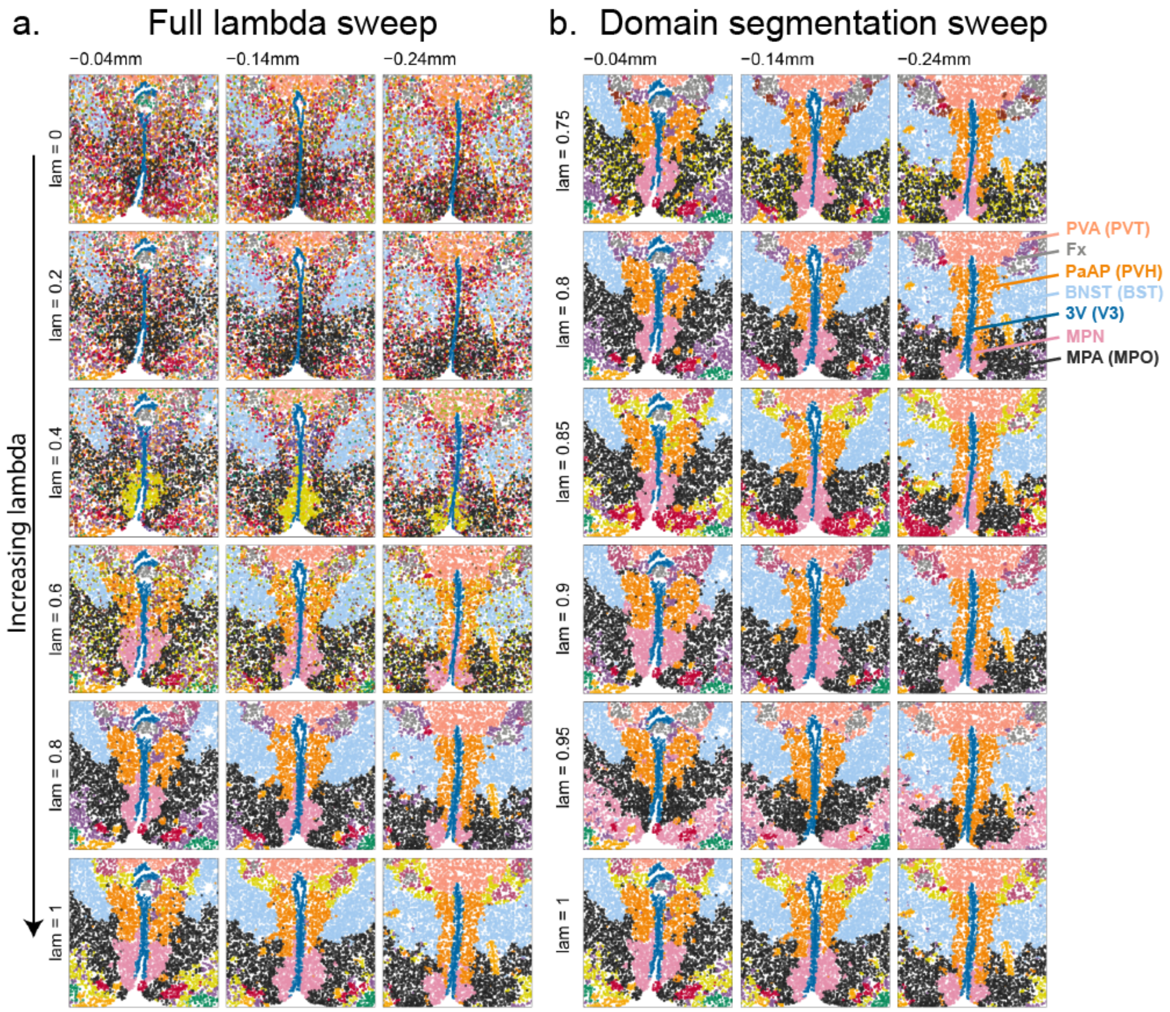

Supp. Fig. 26: Parameter sweep of lambda λ for the MERFISH mouse hypothalamus dataset. (a) Sweep from $\lambda = 0$ (non-spatial), through $\lambda = 0.2$ (cell typing) to $\lambda = 0.8, 1$ (domain segmentation). (b) Domain segmentation lambda sweep in smaller steps. Domain labels as in Fig. 5d.

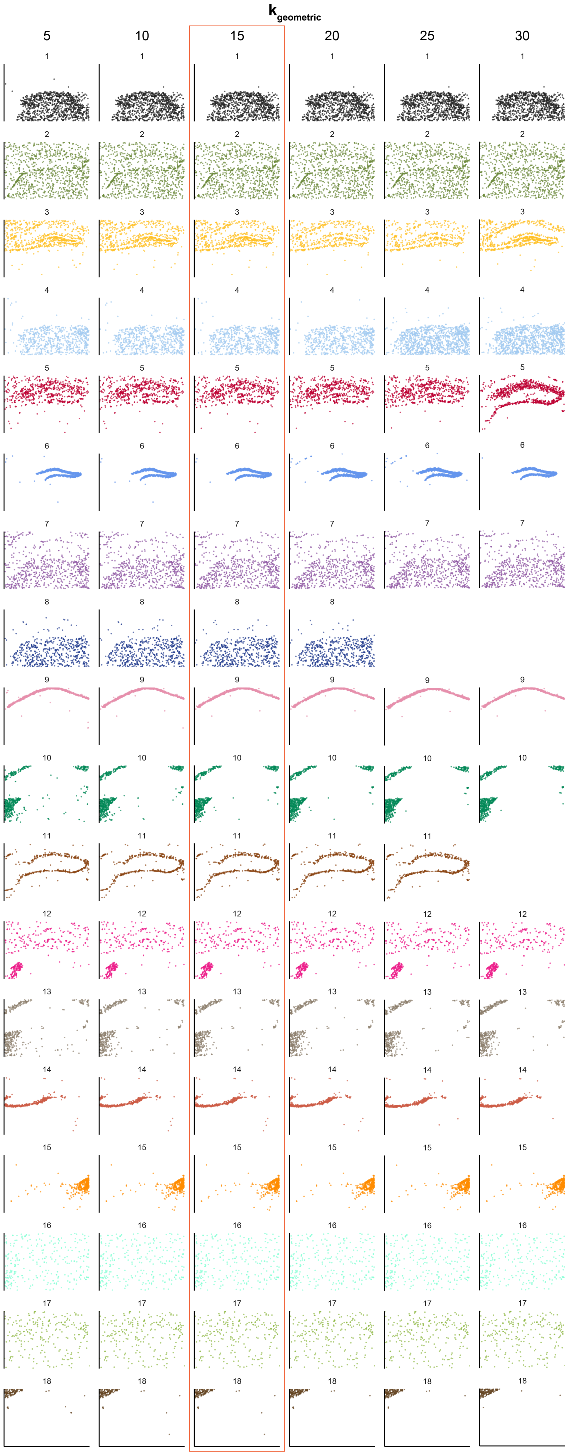

Supp. Fig. 27: Parameter sweep of k_{geom} for VeraFISH mouse hippocampus.

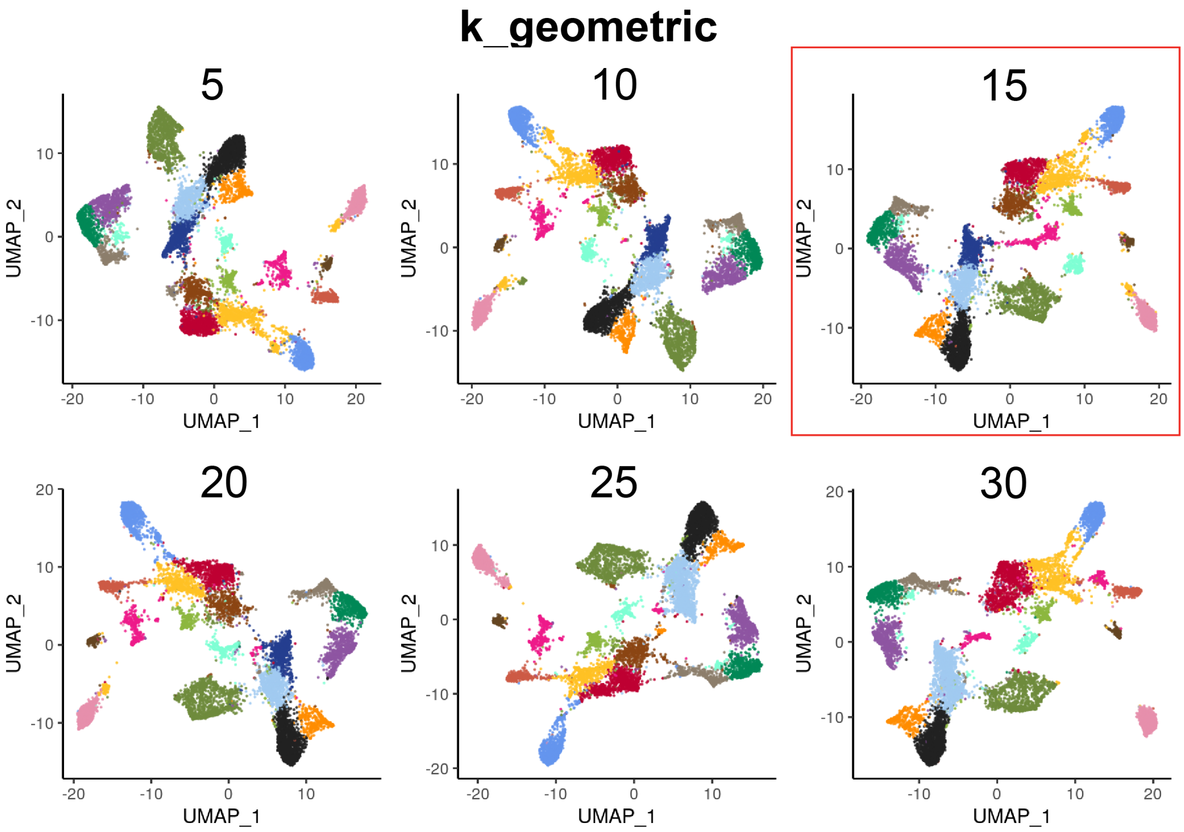

Supp. Fig. 28: Parameter sweep of k_{geom} for VeraFISH mouse hippocampus: UMAPs of the BANKSY embedding.

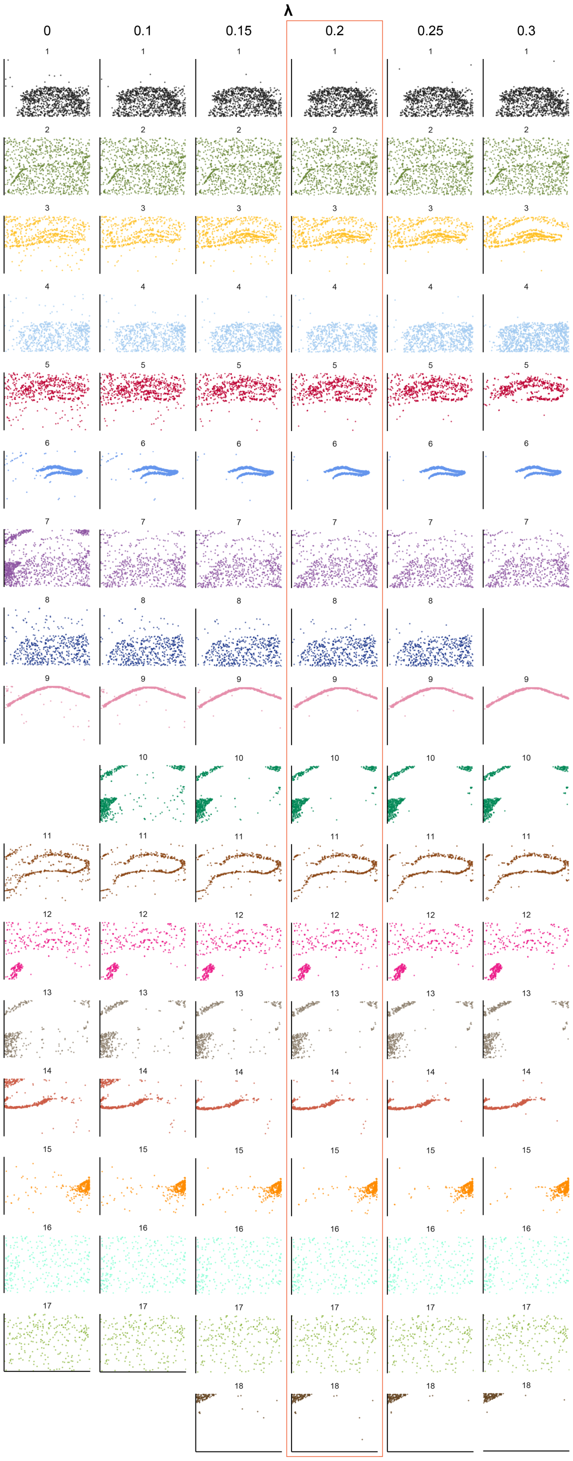

Supp. Fig. 29: Parameter sweep of λ for VeraFISH mouse hippocampus.

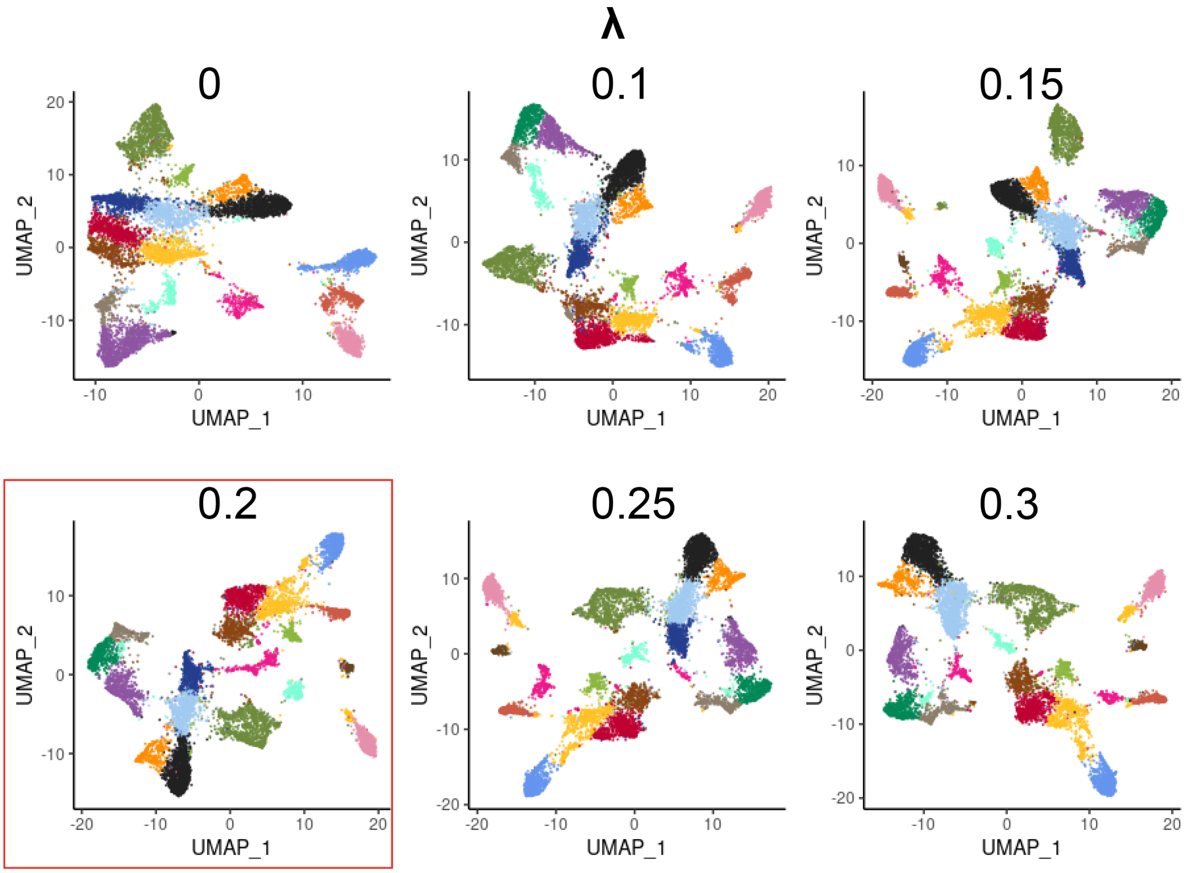

Supp. Fig. 30: Parameter sweep of λ for VeraFISH mouse hippocampus: UMAPs of BANKSY embedding.

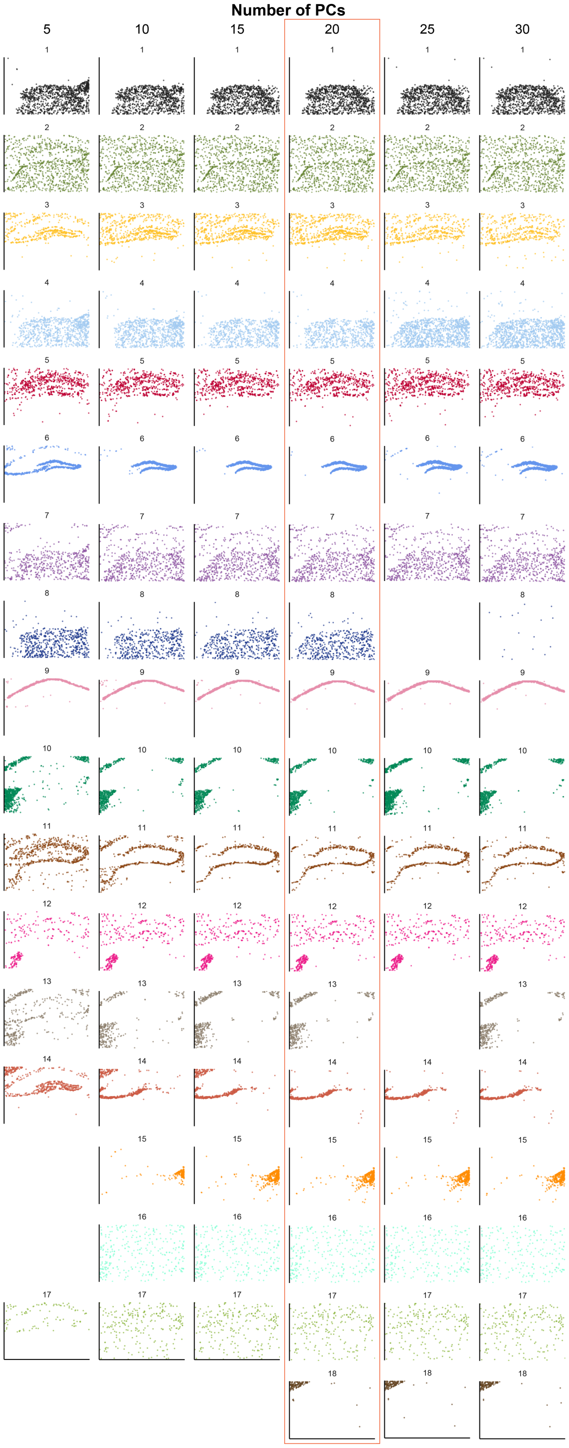

Supp. Fig. 31: Parameter sweep of number of principal components (PCs) for VeraFISH mouse hippocampus.

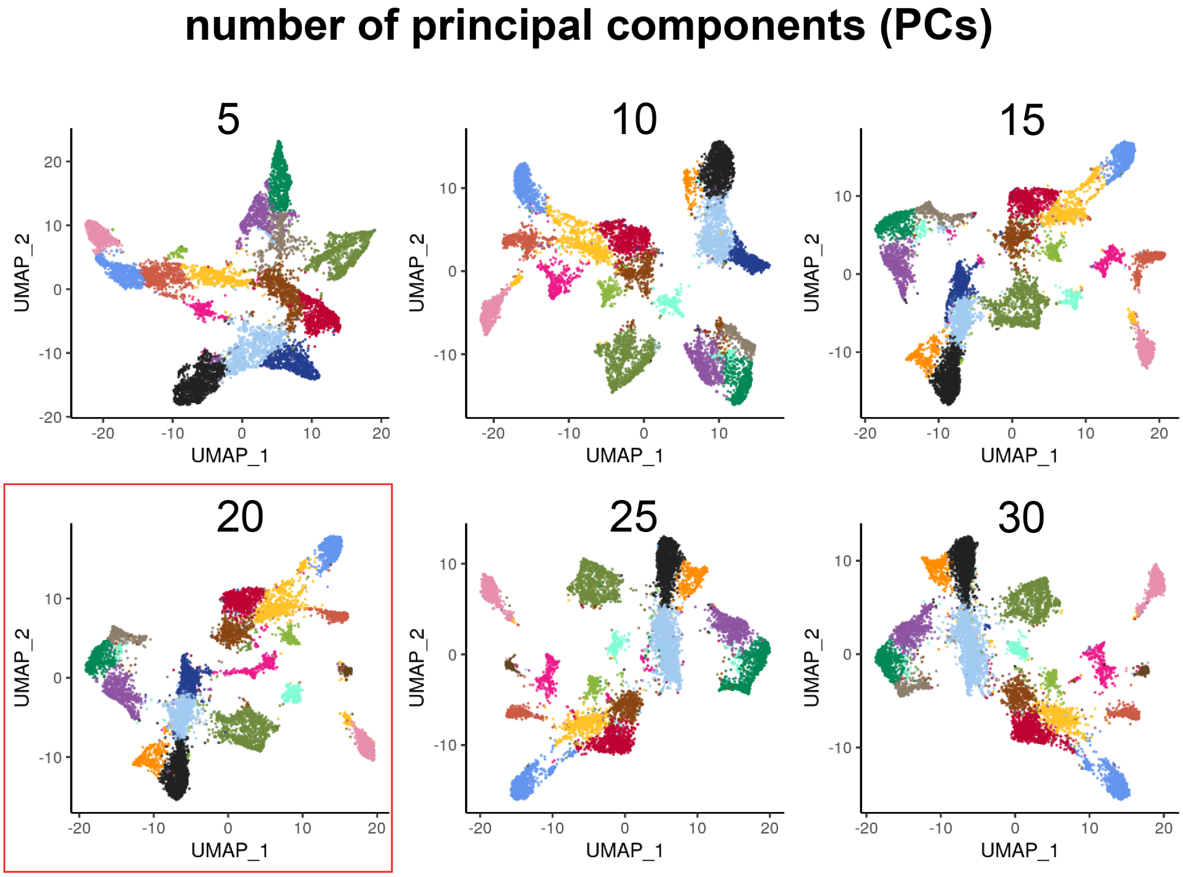

Supp. Fig. 32: Parameter sweep of number of principal components (PCs) for VeraFISH mouse hippocampus: UMAPs of BANKSY embedding.

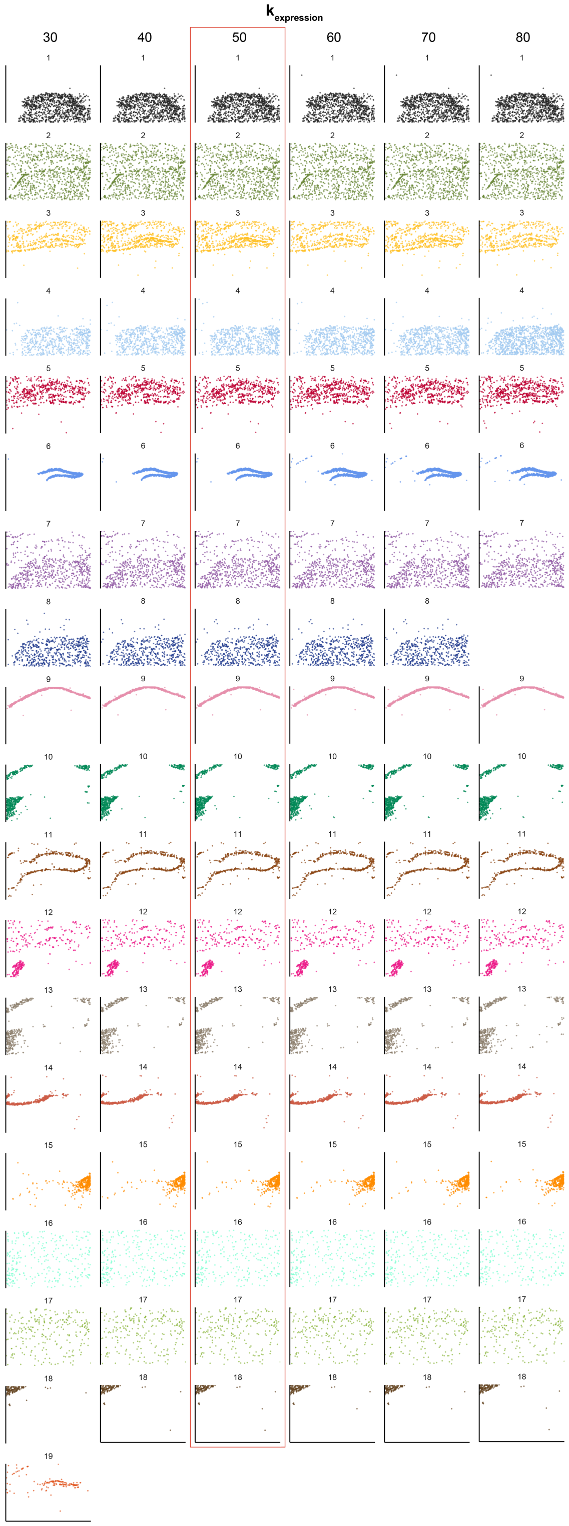

Supp. Fig. 33: Parameter sweep of k_{expr} for VeraFISH mouse hippocampus.

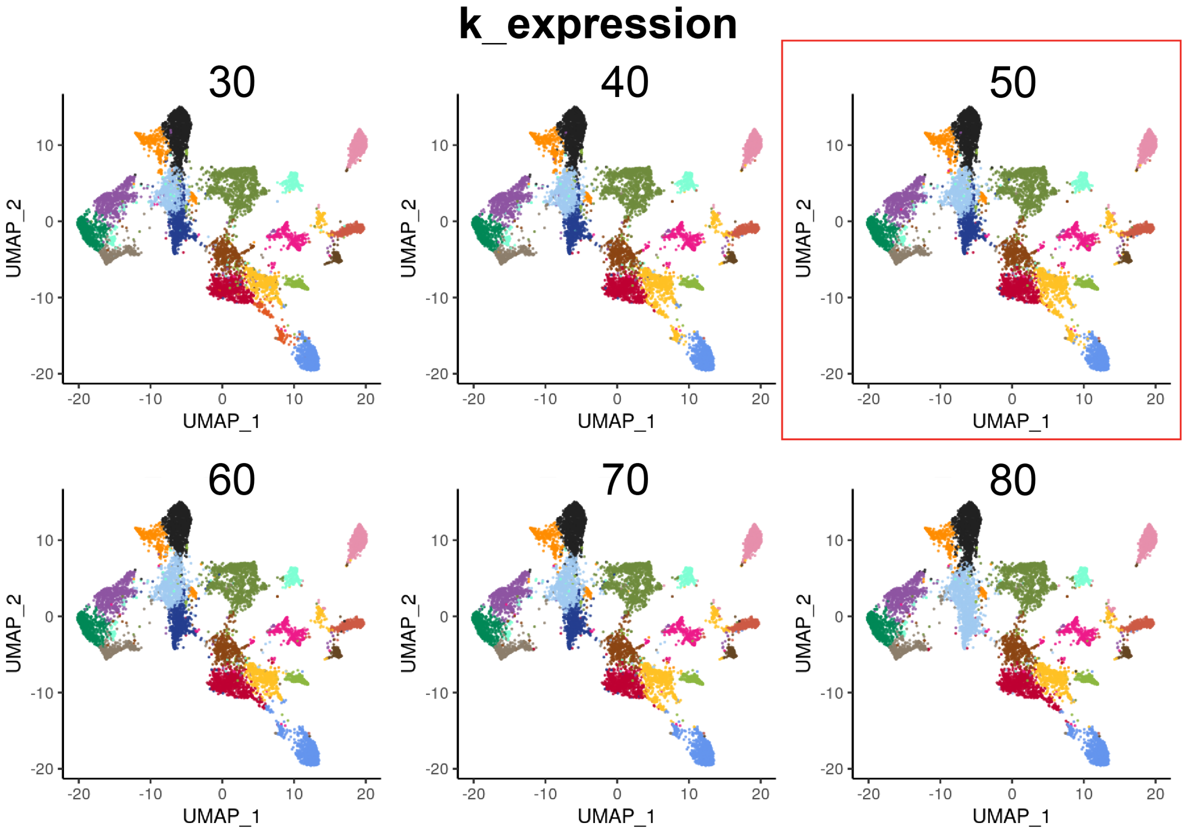

Supp. Fig. 34: Parameter sweep of k_{expr} for VeraFISH mouse hippocampus: UMAPs of BANKSY embedding.

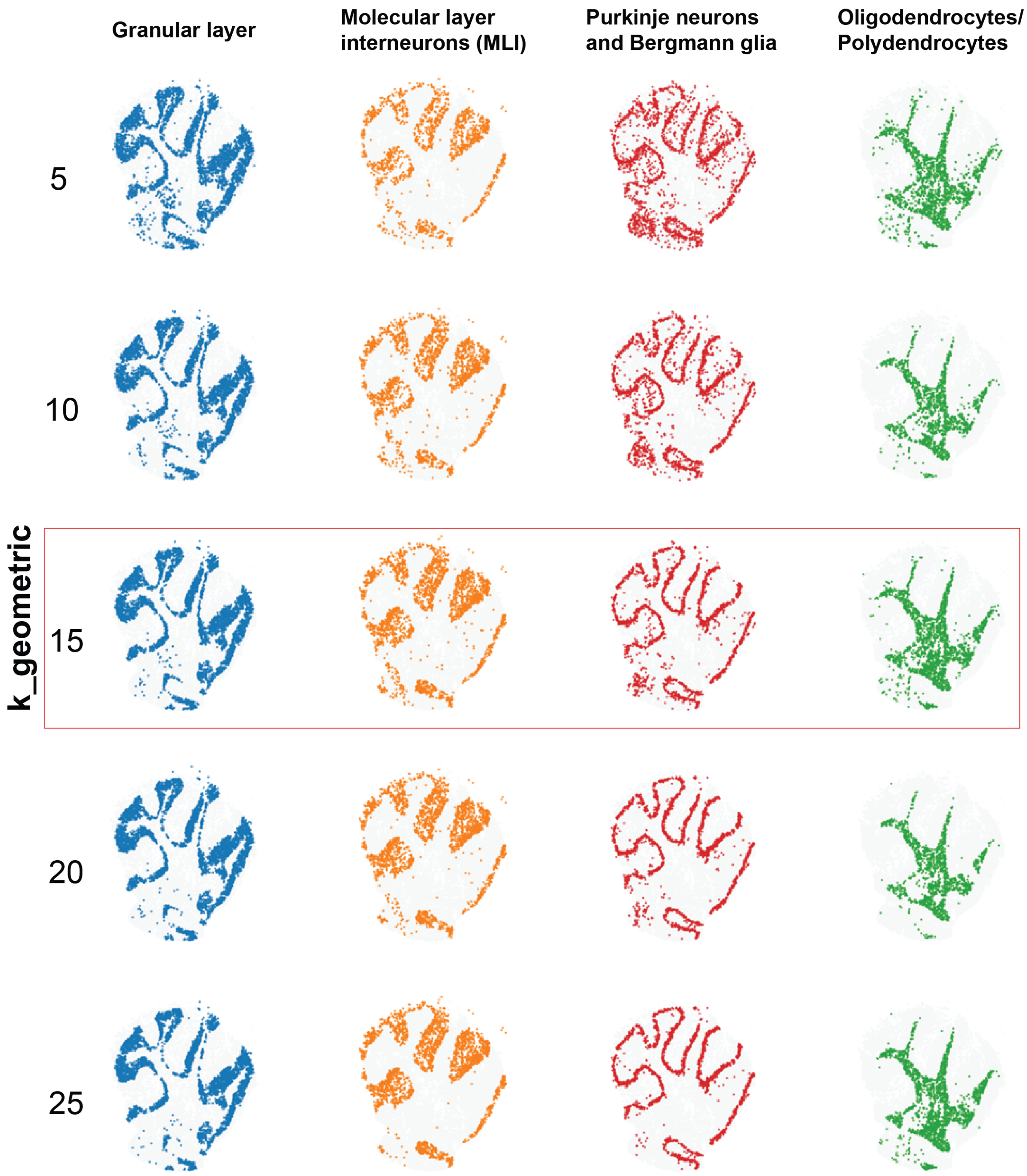

Supp. Fig. 35: Parameter sweep of k_{geom} for Slide-seq v1.

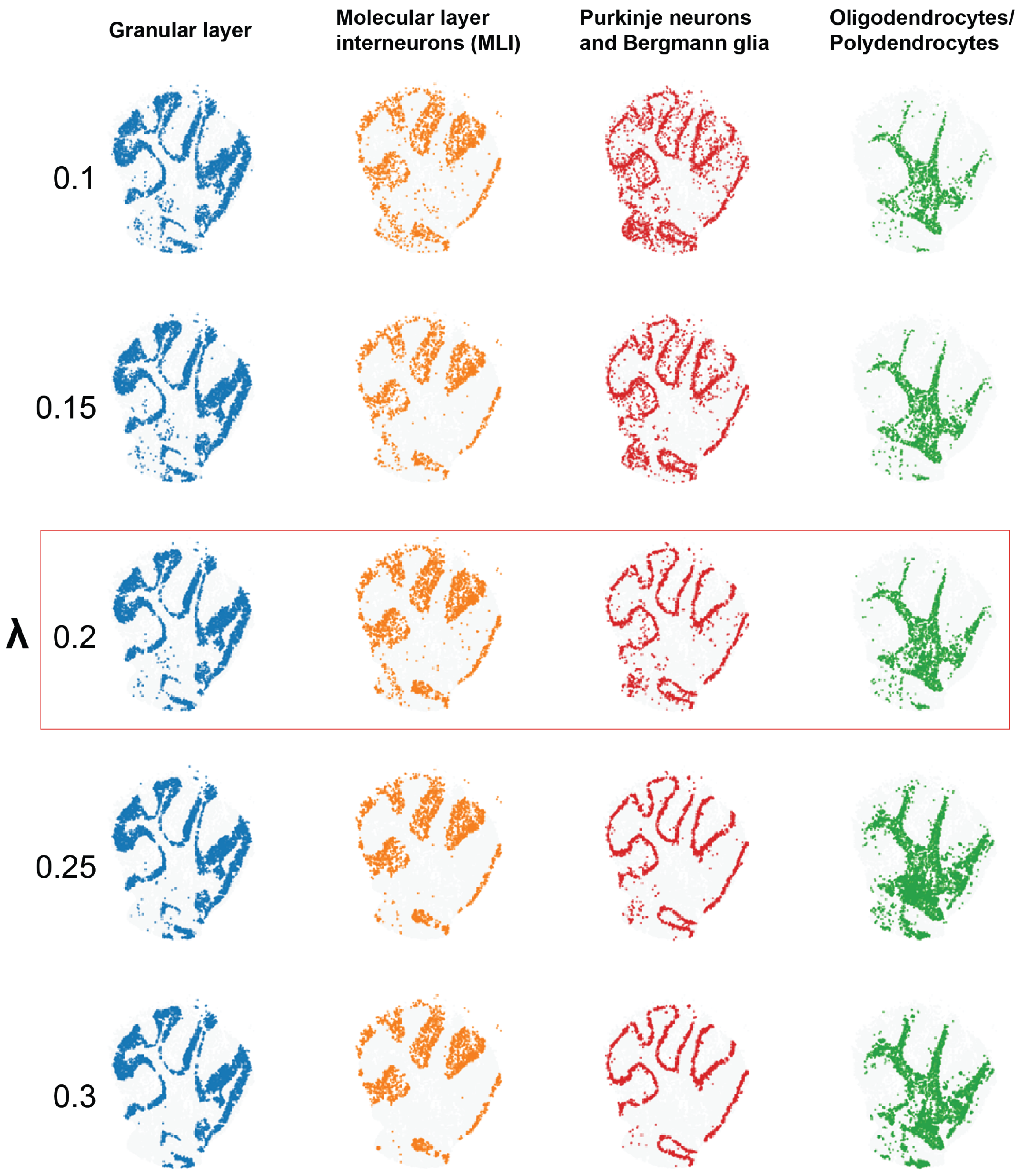

Supp. Fig. 36: Parameter sweep of λ for Slide-seq v1.

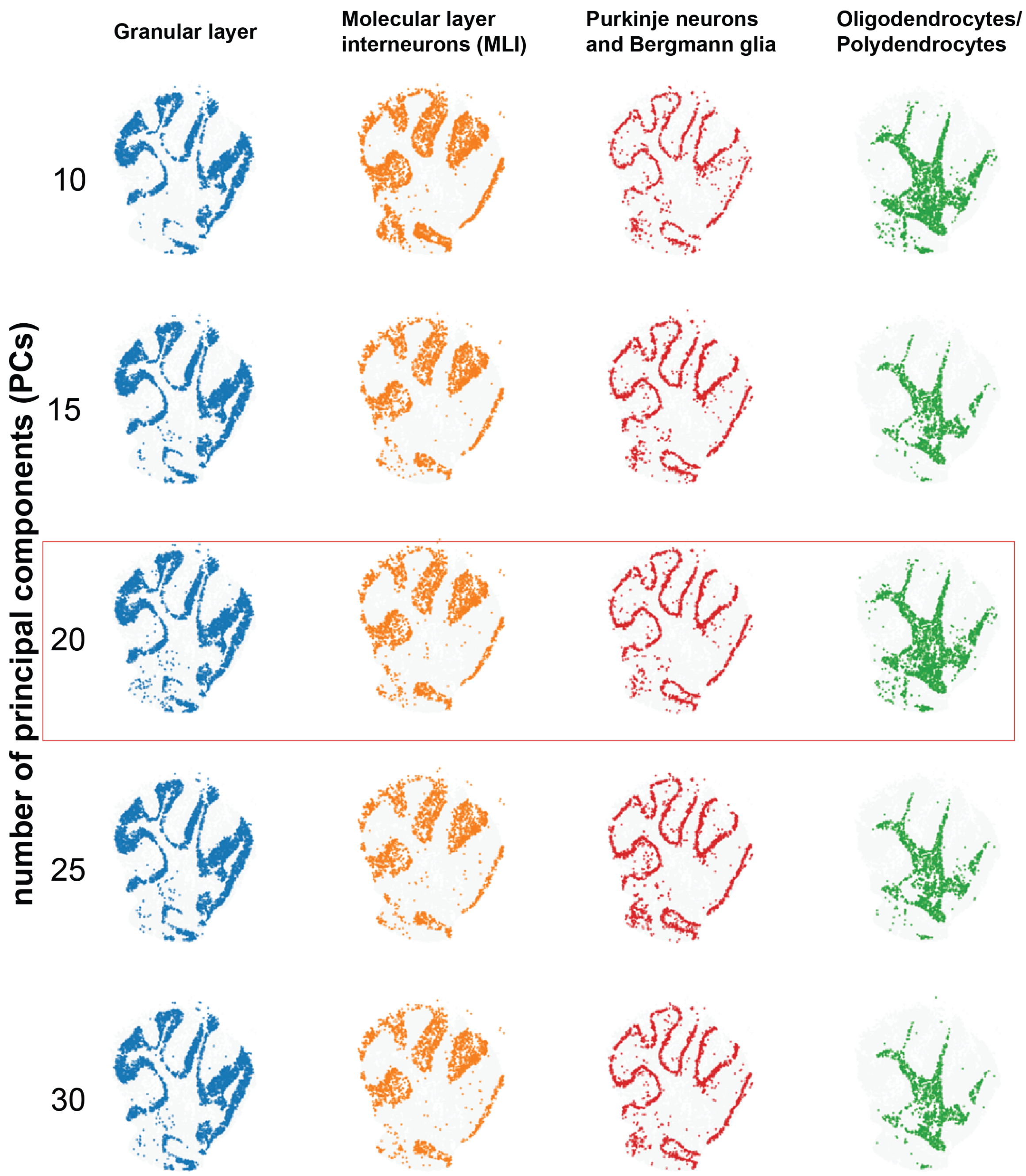

Supp. Fig. 37: Parameter sweep of number of principal components (PCs) for Slide-seq v1.

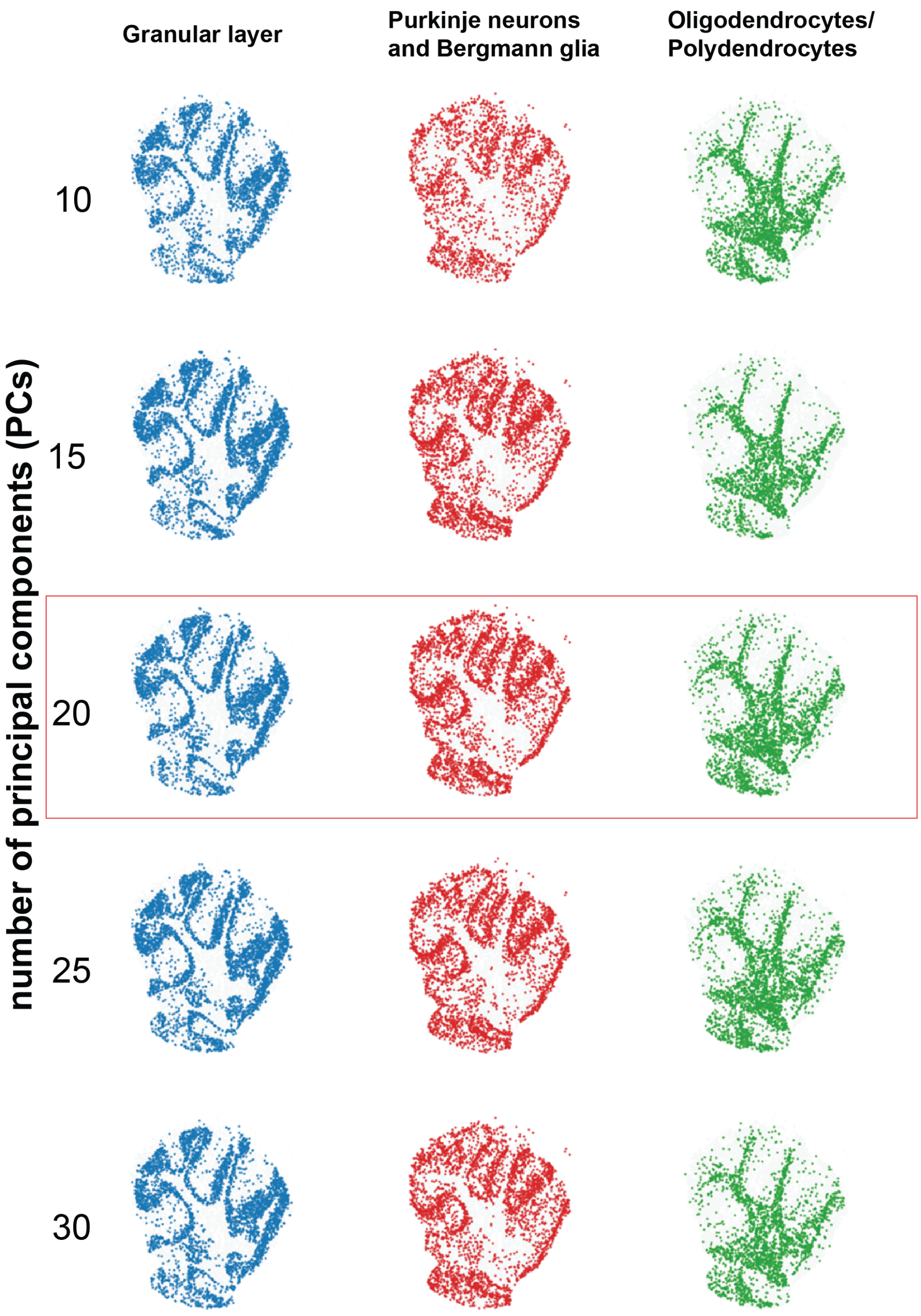

Supp. Fig. 38: Parameter sweep of number of principal components (PCs) for Slide-seq v1 with non-spatial clustering.

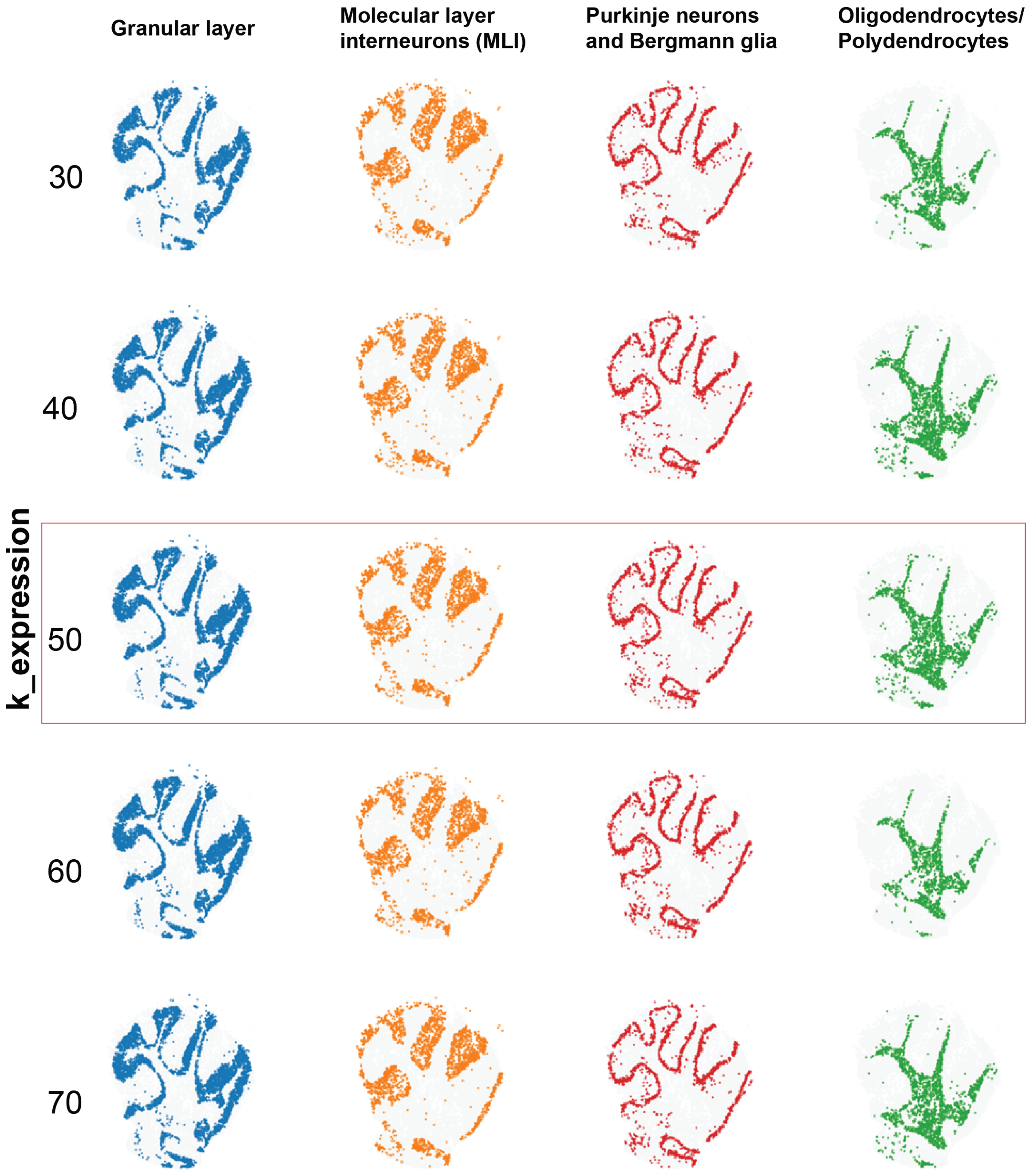

Supp. Fig. 39: Parameter sweep of k_{expr} for Slide-seq v1.

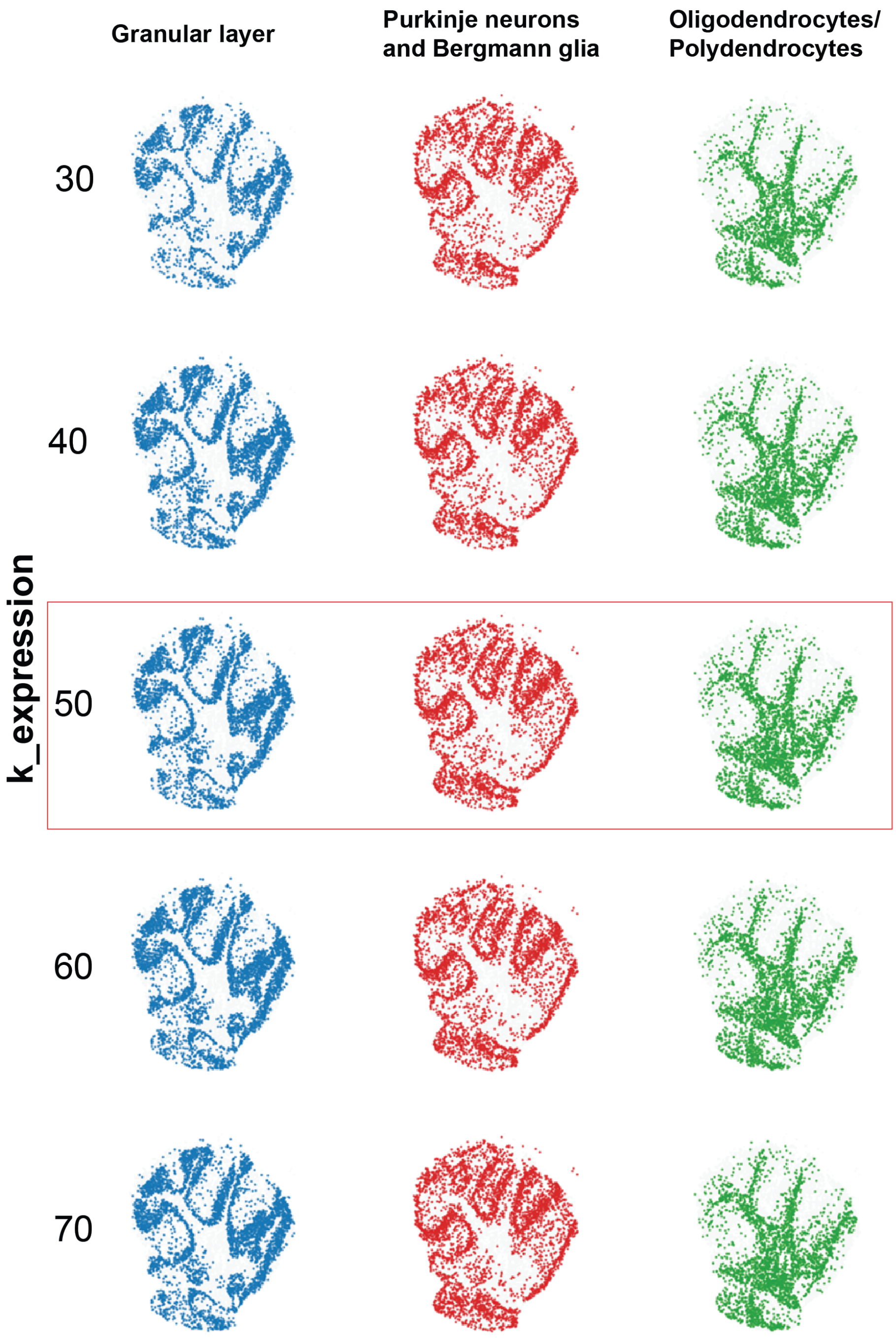

Supp. Fig. 40: Parameter sweep of k_{expr} for Slide-seq v1 with non-spatial clustering.

1 Directly Appending Spatial Coordinates

As mentioned in the Results section, the naive approach of incorporating spatial information into clustering methods is to append the spatial coordinates of cells to their gene expression vectors. This approach was employed by, for instance, the authors of the DLPFC benchmark dataset ([2] Fig. 7f therein). We show that this approach is suboptimal (Supp. Fig. 1) because it assumes that cells that are spatially distant must be from different clusters, which is not true for elongated structures such as epithelial layers and blood vessels, intercalated immune cells resident in tissues and intermingled neuronal and glial cells in the brain.

We describe this approach as follows. As in Section 4.1, let the cells be indexed by the set $\mathcal{U} = \{1, 2, \ldots, N\}$, with spatial coordinates $\mathcal{X} = \{(u, x_u) \in \mathcal{U} \times \mathbb{R}^2\}$. Let us collect the coordinates of the centroids of cells into a matrix,

$$X = \left[\ x_1\ x_2\ \ldots\ x_N\ \right] \in \mathbb{R}^{2\times N}, \tag{1}$$

and as before, we define the gene-cell expression matrix by $\mathcal{C} = \left[\, c_1\ c_2\ \cdots\ c_N \,\right] \in \mathbb{R}^{p\times N}$, where $c_u \in \mathbb{R}^p$ is the expression of the p genes in cell u. We create an augmented feature-cell matrix,

$$A = \begin{bmatrix} \sqrt{1-\lambda}\cdot\mathcal{C} \\ \sqrt{\lambda}\cdot X \end{bmatrix} \in \mathbb{R}^{(p+2)\times N}, \tag{2}$$

which, in terms of distance matrices, is $D_{\text{direct}} = \lambda D_{\text{expression}} + (1-\lambda)D_{\text{physical}}$, where $D_{\text{expression}}$ is the matrix of cell-cell distances in expression space, and D_{physical} is the matrix of pairwise distances in physical space, λ is a mixing parameter that controls the relative weighting of these two types of distances, and D_{direct} is the resulting 'spatially informed' distance matrix in this approach (corresponding to feature-cell matrix A in Equation (2)).

This is similar to the approach taken in the geo-spatial literature [11], where adjacent municipalities in southern France had to be grouped together based on their socio-economic features (analogous to genes in our data), subject to a soft constraint that adjacent municipalities be labelled with the same label.

In spatial omics, this approach does not work because it tends to group cells that are physically near each other into the same cluster, and label physically distant cells differently (Supp. Fig. 1). This is antithetical both to cells of multiple different cell types being interspersed within a single region and to a single cell type being located in different regions of tissue. Supp. Fig. 1 shows the effect of progressively increasing the relative weighting of the spatial coordinates in the example of the two hemispheres of the brain. This section of the brain is symmetric about the midline (for instance, the dentate gyrus, CA1-3 pyramidal neurons, cortical layers, etc., are mirrored along the midline). At small weights, corresponding regions incorrectly receive different labels, although it is still possible for spatially proximal cells to be labeled differently. At higher weights, the tissue labeling degenerates into artefactual and spatially homogeneous 'patches' (Supp. Fig. 1d).

2 Convex Combinations of Squared Dissimilarities

In this section, we show that the weighted concatenation of feature-object matrices can equivalently be formulated as a convex combination of the Hadamard square of corresponding cell-cell dissimilarity matrices computed in BANKSY's component spaces (own expression, mean neighborhood expression, and the AGF features),

$$D_{\mathcal{B}}^{\circ 2} = (1-\lambda)D_{\mathcal{C}}^{\circ 2} + \frac{\lambda}{\mu}D_{\mathcal{M}}^{\circ 2} + \frac{\lambda}{2\mu}D_{\mathcal{G}}^{\circ 2}. \tag{3}$$

Here, $(\,\cdot\,)^{\circ 2}$ denotes element-wise squaring of the matrices, $\{D_{\mathcal{B}}, D_{\mathcal{C}}, D_{\mathcal{M}}, D_{\mathcal{G}}\}$ are dissimilarity matrices corresponding to the embeddings represented by the matrices $\{\mathcal{B}, \mathcal{C}, \mathcal{M}, \mathcal{G}\}$ in Section 4.1, and $\mu = 1.5$ is a normalization factor.

This alternate point of view illuminates a general framework for combining different sources of dissimilarity, or for adding 'soft constraints' [11] into clustering problems. In the BANKSY framework, we are

adding dissimilarity terms associated with the texture of the gene expression in cells' neighborhoods to the traditional transcriptomic dissimilarity used in non-spatial clustering. In principle, other sources of information, such as staining or morphological features could also be included. Furthermore, this alternate formulation allows for the incorporation of (potentially trainable) weights w_{uv} to any of the dissimilarity matrices between cells,

$$D_{(\cdot)} = \begin{bmatrix} w_{11}d^c_{11} & w_{12}d^c_{12} & \cdots & w_{1,N}d^c_{1,N} \\ \vdots & \vdots & \ddots & \vdots \\ w_{N,1}d^c_{N,1} & w_{N,2}d^c_{N,2} & \cdots & w_{N,N}d^c_{N,N} \end{bmatrix}, \tag{4}$$

a property that has no counterpart in the feature-object formulation. In what follows, we describe the equivalence between the two formulations.

Let $\mathcal{B}$, $\mathcal{C}$, $\mathcal{M}$, and $\mathcal{G}$ be as in Section 4.1 and write the uth column of the BANKSY matrix $\mathcal{B}$ as

$$b_i = \begin{bmatrix} \sqrt{1-\lambda}\cdot c_u \\ \sqrt{\lambda/\mu}\cdot h^{\mathcal{M}}_u \\ \sqrt{\lambda/(2\mu)}\cdot h^{\mathcal{G}}_u \end{bmatrix} \in \mathbb{R}^{3p}, \tag{5}$$

where the dot $(\cdot)$ denotes multiplication of a scalar and a vector, c_u, $h^{\mathcal{M}}_u$ and $h^{\mathcal{G}}_u$ are the u-th columns of $\mathcal{C}$, $\mathcal{M}$, and $\mathcal{G}$ respectively.

Define dissimilarity matrices corresponding to the matrices $\{\mathcal{B}, \mathcal{C}, \mathcal{M}, \mathcal{G}\}$,

$$D_{\mathcal{B}} = \begin{bmatrix} d^{\mathcal{B}}_{11} & \cdots & d^{\mathcal{B}}_{1,N} \\ \vdots & \ddots & \vdots \\ d^{\mathcal{B}}_{N,1} & \cdots & d^{\mathcal{B}}_{N,N} \end{bmatrix}, \quad D_{\mathcal{C}} = \begin{bmatrix} d^{\mathcal{C}}_{11} & \cdots & d^{\mathcal{C}}_{1,N} \\ \vdots & \ddots & \vdots \\ d^{\mathcal{C}}_{N,1} & \cdots & d^{\mathcal{C}}_{N,N} \end{bmatrix},$$

$$D_{\mathcal{M}} = \begin{bmatrix} d^{\mathcal{M}}_{11} & \cdots & d^{\mathcal{M}}_{1,N} \\ \vdots & \ddots & \vdots \\ d^{\mathcal{M}}_{N,1} & \cdots & d^{\mathcal{M}}_{N,N} \end{bmatrix}, \quad D_{\mathcal{G}} = \begin{bmatrix} d^{\mathcal{G}}_{11} & \cdots & d^{\mathcal{G}}_{1,N} \\ \vdots & \ddots & \vdots \\ d^{\mathcal{G}}_{N,1} & \cdots & d^{\mathcal{G}}_{N,N} \end{bmatrix},$$

with $d^{\mathcal{B}}_{uv} = \|b_u - b_v\|_2$ the l_2 distances in the neighbor augmented space, $d^{\mathcal{C}}_{uv} = \|c_u - c_v\|_2$ the l_2 distances between cells in expression space, $d^{\mathcal{M}}_{uv} = \|h^M_u - h^M_v\|_2$, and $d^{\mathcal{G}}_{uv} = \|h^G_u - h^G_v\|_2$ the distances between cells in the mean neighborhood expression and AGF spaces.

With these definitions, we may verify (using the properties of the l_2 norm) that,

$$\begin{aligned} (d^{\mathcal{B}})^2_{uv} &= \left\| \begin{bmatrix} \sqrt{1-\lambda}\cdot c_u \\ \sqrt{\lambda/\mu}\cdot h^{\mathcal{M}}_u \\ \sqrt{\lambda/(2^M\mu)}\cdot h^{\mathcal{G}}_u \end{bmatrix} - \begin{bmatrix} \sqrt{1-\lambda}\cdot c_v \\ \sqrt{\lambda/\mu}\cdot h^{\mathcal{M}}_v \\ \sqrt{\lambda/(2^M\mu)}\cdot h^{\mathcal{G}}_v \end{bmatrix} \right\|_2^2 \\ &= (1-\lambda)\|c_u - c_v\|_2^2 + \frac{\lambda}{\mu}\|h^{\mathcal{M}}_u - h^{\mathcal{M}}_v\|_2^2 + \frac{\lambda}{2\mu}\|h^{\mathcal{G}}_u - h^{\mathcal{G}}_v\|_2^2 \\ &= (1-\lambda)(d^{\mathcal{C}}_{uv})^2 + \frac{\lambda}{\mu}(d^{\mathcal{M}}_{uv})^2 + \frac{\lambda}{2\mu}(d^{\mathcal{G}}_{uv})^2 \end{aligned}$$

which is just Equation (3) elementwise.

3 Identification of spatially interspersed cells and rare cell-types

In this section, we explain how BANKSY's feature augmentation strategy allows for the identification of rare and intermingled cell types.

A concern with methods that use neighborhood information to label cells is that the identities of cell-types with subtly differing transcriptomes (e.g., hippocampal astrocytes in Supp. Fig. 17b) or rare cell-types (ependymal cells (2.41% of cells) and microglia (2.12%) in Supp. Fig. 14; M2 macrophages (0.65% of cells) in

Extended Data Fig. 2c, and OPCs (2.28%) and microglia (2.72%) in Supp. Figs. 17c, d) might get 'smoothed out' by the characteristics of their neighbors. This can happen in methods which aggregate a cell's expression with that of its neighbors (for instance, STAGATE, SpaGCN and GraphST). It can also happen implicitly with HMRF-based methods that use the Potts model for regularization (for instance, BayesSpace and Giotto) to bias the label given to a cell by the labels given to its neighbors. Both these classes of methods tend to label cells based on the average or majority identity of cells in each neighborhood, and as a result, are better suited to domain segmentation than cell-typing.

BANKSY takes a different approach. Instead of averaging a cell's expression with the average (and AGF) of its neighbors, it *concatenates* the three sets of features to give a larger set that together defines that cell's identity. This lifts the cells to a higher dimensional space where the unique characteristics of a cell's own expression and that of its neighborhood (i.e., average and AGF) are maintained separately.

The intuition for why such lifting works is best illustrated using the schematic shown in Extended Data Fig. 1b. The middle panel in the bottom row shows what BANKSY's neighbor-augmented space looks like in cell-typing mode: the own expression and neighborhood expressions are along separate axes, and cells may be separated along either or both axes.

In the case of two intermingled cell types with subtly different expression signatures, the cells' neighborhood expressions (encoded by average and AGF) are almost identical (they lie close to each other along the red vertical axis), but their own expression signatures are different (they lie at different points along the purple horizontal axis), and this separation in the embedding space can be used by the clustering algorithm to assign them different labels. Similarly, in the case of a rare cell type surrounded by cells of other types, the rare cell and its neighbors have identical neighborhood transcriptomes (similar points on the vertical axis) but different own expression signatures. In both scenarios, BANKSY maintains the own expression signature as a separate set of axes from the neighborhood expression signature, a property that is not present in averaging based methods.

As mentioned above, averaging or aggregating a cell's expression with that of its neighbors results in an embedding that reflects local averages of cells' transcriptomes. This property enables the clustering algorithm to group cells into spatial domains defined by local transcriptomic characteristics. BANKSY achieves domain segmentation in a similar way (rightmost panel, bottom row of Extended Data Fig. 1) by weighting the neighborhood features more heavily, such that the embedding is dominated by the local neighborhood averages.

References

[1] Vizgen Data Release Program. https://vizgen.com/support/data-release-program, May 2021.

[2] Kristen R. Maynard, Leonardo Collado-Torres, Lukas M. Weber, Cedric Uytingco, Brianna K. Barry, Stephen R. Williams, Joseph L. Catallini, Matthew N. Tran, Zachary Besich, Madhavi Tippani, Jennifer Chew, Yifeng Yin, Joel E. Kleinman, Thomas M. Hyde, Nikhil Rao, Stephanie C. Hicks, Keri Martinowich, and Andrew E. Jaffe. Transcriptome-scale spatial gene expression in the human dorsolateral prefrontal cortex. Nature Neuroscience, 24(3):425–436, March 2021.

[3] Jeffrey R. Moffitt, Dhananjay Bambah-Mukku, Stephen W. Eichhorn, Eric Vaughn, Karthik Shekhar, Julio D. Perez, Nimrod D. Rubinstein, Junjie Hao, Aviv Regev, Catherine Dulac, and Xiaowei Zhuang. Molecular, spatial, and functional single-cell profiling of the hypothalamic preoptic region. Science, 362(6416), November 2018.

[4] Florian Duclot and Mohamed Kabbaj. The Role of Early Growth Response 1 (EGR1) in Brain Plasticity and Neuropsychiatric Disorders. Frontiers in Behavioral Neuroscience, 11:35, March 2017.

[5] Baljit S. Khakh and Michael V. Sofroniew. Diversity of astrocyte functions and phenotypes in neural circuits. Nature neuroscience, 18(7):942–952, July 2015.

[6] Brain tissue expression of GFAP - Mouse brain - The Human Protein Atlas. https://www.proteinatlas.org/ENSG00000131095-GFAP/brain/primary+mouse+data.

[7] Lisa A. Thomas, Michael R. Akins, and Thomas Biederer. Expression and Adhesion Profiles of SynCAM Adhesion Molecules Indicate Distinct Neuronal Functions. The Journal of comparative neurology, 510(1):47–67, September 2008.

[8] Pyramidal cells gene expression markers | PanglaoDB. https://panglaodb.se/markers.html?cell%20type=%27Pyramidal%20cells%27.

[9] Allen Mouse Brain Atlas [dataset]. http://mouse.brain-map.org, 2011.

[10] Ed S. Lein et al. Genome-wide atlas of gene expression in the adult mouse brain. Nature, 445(7124):168–176, January 2007.

[11] Marie Chavent, Vanessa Kuentz-Simonet, Amaury Labenne, and Jérôme Saracco. ClustGeo: an R package for hierarchical clustering with spatial constraints. Computational Statistics, 33(4):1799–1822, December 2018. arXiv: 1707.03897.
