# Supplementary material for: BANKSY unifies cell typing and tissue domain segmentation for scalable spatial omics data analysis
Source: Nat Genet. 2024 Feb 27;56(3):431–41. doi: 10.1038/s41588-024-01664-3 (PMC10937399; doi:10.1038/s41588-024-01664-3)
Supplement: Supplementary file 2 — Reporting Summary [file 41588_2024_1664_MOESM2_ESM.pdf]

nature portfolio

Corresponding author(s): Shyam Prabhakar and Kok Hao Chen

Last updated by author(s): Dec 4, 2023

Reporting Summary

Nature Portfolio wishes to improve the reproducibility of the work that we publish. This form provides structure for consistency and transparency in reporting. For further information on Nature Portfolio policies, see our Editorial Policies and the Editorial Policy Checklist.

Statistics

For all statistical analyses, confirm that the following items are present in the figure legend, table legend, main text, or Methods section.

n/a	Confirmed	
☐	☒	The exact sample size (*n*) for each experimental group/condition, given as a discrete number and unit of measurement
☒	☐	A statement on whether measurements were taken from distinct samples or whether the same sample was measured repeatedly
☐	☒	The statistical test(s) used AND whether they are one- or two-sided<br>*Only common tests should be described solely by name; describe more complex techniques in the Methods section.*
☒	☐	A description of all covariates tested
☒	☐	A description of any assumptions or corrections, such as tests of normality and adjustment for multiple comparisons
☐	☒	A full description of the statistical parameters including central tendency (e.g. means) or other basic estimates (e.g. regression coefficient) AND variation (e.g. standard deviation) or associated estimates of uncertainty (e.g. confidence intervals)
☐	☒	For null hypothesis testing, the test statistic (e.g. *F*, *t*, *r*) with confidence intervals, effect sizes, degrees of freedom and *P* value noted<br>*Give P values as exact values whenever suitable.*
☐	☒	For Bayesian analysis, information on the choice of priors and Markov chain Monte Carlo settings
☒	☐	For hierarchical and complex designs, identification of the appropriate level for tests and full reporting of outcomes
☐	☒	Estimates of effect sizes (e.g. Cohen's *d*, Pearson's *r*), indicating how they were calculated

Our web collection on statistics for biologists contains articles on many of the points above.

Software and code

Policy information about availability of computer code

Data collection	The VeraFISH data was collected using the VSA-1 Imager from Veranome Biosystems, and the collected images were processed throught the VeraWorks software (version 1.0_006) to reconstruct the locations of the barcode probes. Segmentation was performed using a Mask-RCNN neural network, also part of the same software.
Data analysis	All BANKSY analysis was done with the Python (Python version 3.8, BANKSY version 1.1.0) or R (R version 4 (version >3.5), BANKSY version 0.1.5). For solving the linear sum assignment problem via the Hungarian algorithm, we used scipy (version 1.6.2) in the Python version of BANKSY, and the RcppHungarian (version 0.3) in the R version. The Seurat analysis of the VeraFISH and MERFISH data was done with Seurat version 4.1.1. Other software versions were as follows: BayesSpace version 1.5.1, Spicemix latest version (Git commit id: aea69f8), Leiden clustering from igraph version 1.2.11, scran version 1.18.7, SingleR version 1.4.1, Nebulosa version 1.0.2, SpaGCN version 1.2.2, Giotto version 1.1.0, BayesSpace version 1.5.1, STAGATE version 1.0.1, GraphST version 1.1.1, Harmony version 0.1.1, scDesign2 version 0.1.0, FICT version 1.0.0, MERINGUE version 1.0, peakRAM version 1.0.2 and CUDA version 12.0.<br><br>The R package, as well as scripts to reproduce analyses, can be obtained from https://github.com/prabhakarlab/Banksy, while the python version is available from https://github.com/prabhakarlab/Banksy_py. The IPython notebooks to reproduce our analysis on Slide-seq v1, Slide-seq v2 and STARmap are available on the Banksy-manscuript branch of the BANKSY_py Github repository. The scripts to reproduce the R analysis are available via https://github.com/jleechung/banksy-zenodo and via the Zenodo file repository: 10.5281/zenodo.1025879

For manuscripts utilizing custom algorithms or software that are central to the research but not yet described in published literature, software must be made available to editors and reviewers. We strongly encourage code deposition in a community repository (e.g. GitHub). See the Nature Portfolio guidelines for submitting code & software for further information.

Data

Policy information about availability of data

All manuscripts must include a data availability statement. This statement should provide the following information, where applicable:

- Accession codes, unique identifiers, or web links for publicly available datasets
- A description of any restrictions on data availability
- For clinical datasets or third party data, please ensure that the statement adheres to our policy

The SlideSeq mouse cerebellum data were obtained from https://github.com/RubD/spatial-datasets/blob/ master/data/2019_slideseq_cerebellum/raw_data/slideseq_cerebellum_urls.txt. We used the BeadLocationsForR and MappedDGEForR .csv files. For Slide-seq V2, we obtained the data from the Broad Institute Single Cell Portal at https://singlecell.broadinstitute.org/single_cell/study/SCP948.The MERFISH mouse hypothalamus data were obtained from https://doi.org/10.5061/dryad.8t8s248. The Vizgen MERSCOPE data for sample `Colon cancer 1' was obtained from https://info.vizgen.com/ffpe-showcase. The CosMX SMI data of the human healthy colon was obtained from NCBI GEO database (accession GSM7473683). The processed CODEX multiplexed imaging data of the healthy human intestine were downloaded from https://datadryad.org/stash/dataset/doi:10.5061/dryad.pk0p2ngrf. The mouse hippocampus data were collected using the VeraFISH assay (Veranome Biosystems, LLC, Mountain View, CA, USA) as described in Section 8.7. The data are available by running the command data(hippocampus) in the R version of the BANKSY package or directly from https://github.com/prabhakarlab/Banksy/blob/bioc/data/hippocampus.rda. The 10x Visium data of the dorsolateral prefrontal cortex (DLPFC) were obtained from the spatialLIBD project (http://spatial.libd.org/spatialLIBD) (Pardo B., et al., 2022). The STARmap mouse visual cortex data were obtained from http://clarityresourcecenter.org.

Research involving human participants, their data, or biological material

Policy information about studies with human participants or human data. See also policy information about sex, gender (identity/presentation), and sexual orientation and race, ethnicity and racism.

Reporting on sex and gender	No human data were collected as part of the study.
Reporting on race, ethnicity, or other socially relevant groupings	No human data were collected as part of the study.
Population characteristics	No human data were collected as part of the study.
Recruitment	No human data were collected as part of the study.
Ethics oversight	No human data were collected as part of the study.

Note that full information on the approval of the study protocol must also be provided in the manuscript.

Field-specific reporting

Please select the one below that is the best fit for your research. If you are not sure, read the appropriate sections before making your selection.

☒ Life sciences ☐ Behavioural & social sciences ☐ Ecological, evolutionary & environmental sciences

For a reference copy of the document with all sections, see nature.com/documents/nr-reporting-summary-flat.pdf

Life sciences study design

All studies must disclose on these points even when the disclosure is negative.

Sample size	In the Slide-seq and Slide-seq v2 datasets, we used all cells (25,551 and 39496 cells respectively). In the MERFISH mouse hypothalamus data (Fig. 3a-e), all 11 Naive animals were used for analysis (485,657 cells). In the scRNA-seq study of mature oligodendrocytes in the mouse hypothalamus (Fig. 3f), all cells labeled 'Mature Oligodendrocytes' by the authors of the original study were used after standard QC cutoffs (cells with >20% mitochondrial genes or <1000 NODG, see Methods), resulting in 6611 cells. In the VeraFISH mouse hippocampus dataset (Fig. 4a-r), we used all 10,994 cells in the dataset. For the corresponding scRNA-seq analysis, all cells with neuronal clusters corresponding to the relevant cortical and hippocampad CA3 regions (as labeled by the authors of the original study) were used, resulting in 2386 cells. Similarly, all cells labeled 'Oligo' were used for the oligodendrocyte analysis, resulting in 231 cells. In the MERSCOPE CRC data (Fig. 3f-h), we used all 677,451 cells in the dataset. In the DLPFC Visium dataset we used all 12 samples in the dataset, as is standard in the benchmarking performed in the field. For the STARMAP dataset, we used all 1207 cells annotated by the authors. In the colon CosMx data, there were two major connected (contiguous) regions of fields of view (FOVs), along with some FOVs with scattered cells. We used the larger of these connected regions for analysis, resulting in 32,765 cells. In the CODEX data, we used all 33,958 cells from the ileum, all 25,403 cells from the right color region, and all 27,784 cells from the transverse colon region for tissue domain annotation from Donor B0012 and 38371 cells from from donor B006 in the ascending colon region for community annotation. In the simulated data, we generated datasets modeled of real STARMAP data (see Methods Section 8.15), and created 3 samples simulated for each gene-set condition (400, 600, 800 and 1020 genes), comprising 4996 cells each.
Data exclusions	In the mouse hypothalamus MERFISH data, we followed the authors of the original study to remove cells marked 'Ambiguous' by them and the gene Fos, which contained 'NaN' entries. In the DLPFC Visium data, we removed spots marked 'ambiguous' by the authors. In the CosMx

data, there two major connected regions of FOVs, along with some FOVs with scattered cells. We used the larger of these connected regions for analysis, discarding the smaller region and the FOVs with scattered cells. In all other analyses, we used all cells, and applied standard QC cutoffs.

Replication	The DLPFC data comprises 4 samples from each of 3 patients, resulting in 12 total datasets. following the usual practice in the field, we reported median statistics and boxplots over these 12 samples. For the simulated data, we generated 3 replicates for each gene count condition (400, 600, 800, all 1020) and reported median values of ARIs over these replicated for all tested methods. In all other datasets, the entire dataset was clustered and analyzed as a single dataset.
Randomization	In the simulated dataset, the genes to subset were picked randomly to generate 400, 600, 800 gene sets of the full 1020 gene set. No other randomization was used in this study (deterministic subsetting methods like highly variable genes were used for gene selection, and quality control metrics like number of detected genes were used for cell subsetting).
Blinding	Blinding was not applicable to this study because no sample group allocation was performed.

Reporting for specific materials, systems and methods

We require information from authors about some types of materials, experimental systems and methods used in many studies. Here, indicate whether each material, system or method listed is relevant to your study. If you are not sure if a list item applies to your research, read the appropriate section before selecting a response.

Materials & experimental systems

n/a	Involved in the study	
☒	☐	Antibodies
☒	☐	Eukaryotic cell lines
☒	☐	Palaeontology and archaeology
☐	☒	Animals and other organisms
☒	☐	Clinical data
☒	☐	Dual use research of concern
☒	☐	Plants

Methods

n/a	Involved in the study	
☒	☐	ChIP-seq
☒	☐	Flow cytometry
☒	☐	MRI-based neuroimaging

Animals and other research organisms

Policy information about studies involving animals; ARRIVE guidelines recommended for reporting animal research, and Sex and Gender in Research

Laboratory animals	One six weeks old female mouse (C57BL/6NTac) was purchased from InVivos, Singapore, (https://www.invivos.com.sg/c57bl-6ntac/#tab-60391), and was euthanized and dissected for removal of the brain immediately upon receipt (the mice were not housed and housing conditions do not apply).
Wild animals	No wild animals were used in the study.
Reporting on sex	Sex differences were not the aim of this study. Mouse brain data was only used to compare different spatial clustering algorithms.
Field-collected samples	No field collected samples were used for this study.
Ethics oversight	All animal procedures were done in accordance with the approved Institutional Animal Care and Use Committee (IACUC) protocol (Protocol #211580) obtained from the IACUC of the biomedical resource center.

Note that full information on the approval of the study protocol must also be provided in the manuscript.

Plants

Seed stocks	No plant data was collected or analyzed as part of this study.
Novel plant genotypes	No plant data was collected or analyzed as part of this study.
Authentication	No plant data was collected or analyzed as part of this study.
